# Shared striatal neurons exhibit context-specific dynamics for internally and externally driven actions

**DOI:** 10.1126/sciadv.aed9386

**Published:** 2026-07-29

**Authors:** Jan L. Klee, Sulekh Fernando-Peiris, Sahil Suresh, Ines Rodrigues-Vaz, Darcy S. Peterka, Rui M. Costa, Vivek R. Athalye, Tanya Sippy

**Affiliations:** ^1^ Department of Psychiatry and Neuroscience, New York University Grossman School of Medicine, New York, NY 10016, USA.; ^2^ Allen Institute, Allen Institute for Neural Dynamics, Seattle, WA 98109, USA.; ^3^ Zuckerman Mind Brain Behavior Institute, Departments of Neuroscience and Neurology, Columbia University, New York, NY 10027, USA.

## Abstract

Animals can initiate movements either in response to external cues or from internal drive, yet how the brain flexibly supports both remains unclear. Disorders such as Parkinson’s disease disrupt these modes differently, suggesting distinct underlying mechanisms. These differences could arise from specialized circuits or from shared neuronal populations that shift their dynamics across contexts. To distinguish between these possibilities, we performed two-photon calcium imaging in the dorsolateral striatum as mice executed the same lever press either spontaneously or in response to a cue. Unsupervised clustering identified neurons modulated during cue, movement, or postaction periods. Critically, the same neurons encoded movement across initiation contexts, but their population dynamics diverged before movement. Both D1- and D2-expressing spiny projection neurons contributed to these dynamics, with D1-SPNs more active at the time of the sensory stimulus. These results show that context shapes neural dynamics within a shared movement-encoding population, revealing a context-generalizable striatal code that supports flexible movement initiation across internal and external drives.

## Teaser

Shared striatal neurons differentiate self-generated and cue-driven actions through distinct activity patterns.

## INTRODUCTION

Understanding the mechanisms that distinguish self-initiated from sensory-evoked movements is essential for elucidating the neural basis of motor control. This distinction has clinical relevance: Patients with Parkinson’s disease often experience difficulty initiating voluntary movements yet can transiently regain motor function in response to external cues such as visual or auditory stimuli (*1*, *2*). These observations suggest that sensory-guided movements engage neural circuits that are at least partially distinct from those underlying internally generated actions or, alternatively, may modulate activity within shared motor circuits to bias movement initiation. At the cortical level, self-evoked and externally triggered actions are represented by largely distinct neural ensembles (*3*–*5*)—for example, sensory cortical neurons are preferentially recruited by external cues, whereas medial frontal neurons show ramping activity before self-initiated movements (*5*). However, recent population-level analyses point to a shared preparatory component within these areas, indicating that overlapping neuronal populations can support both initiation modes (*6*). More broadly, prior work has demonstrated that cortical circuits often embed multiple task variables within shared low-dimensional subspaces (*7*, *8*), suggesting that mixed selectivity and population-level organization may allow flexible routing of both self-initiated and stimulus-driven signals.

The striatum, the input nucleus of the basal ganglia, integrates information from widespread cortical and subcortical sources (*9*–*13*), enabling context-dependent initiation of the same action (*14*, *15*). Its cortical and subcortical inputs are topographically organized: Anterior regions of the dorsal striatum are predominantly innervated by motor areas, whereas more posterior regions receive dense input from sensory areas, including visual, somatosensory, and auditory cortex (*12*, *16*–*18*). In the dorsolateral striatum (DLS), individual neurons are thought to receive convergent input from both sensory and motor cortical areas (*19*–*21*), positioning them to integrate contextual and motor-related signals. Consistent with this, single-unit recordings in nonhuman primates demonstrated that subsets of striatal neurons are preferentially engaged during either self-initiated or stimulus-driven movements, while only a minority show overlapping activity across both contexts (*22*).

Striatal projection neurons (SPNs) are broadly divided into two major classes based on their dopamine receptor expression: D1-SPNs, which form the direct pathway and are classically associated with promoting action initiation, and D2-SPNs, which form the indirect pathway and are linked to action suppression (*23*). While these pathways have traditionally been viewed as functionally opposing, accumulating evidence indicates that both are active during movement and may contribute in complementary ways to shaping behavior (*24*–*27*). Recent work from our group and others has shown that in sensory-driven behaviors, D1-SPNs often exhibit stronger and more time-locked activation to external cues (*28*–*32*), suggesting that the direct pathway may be particularly responsive to salient sensory events that trigger action.

However, it remains unclear how these inputs are funneled to drive action in the appropriate context, and how D1- and D2-SPNs may differentially contribute to this process. Do distinct contextual demands recruit nonoverlapping neuronal populations, or do shared neurons integrate different inputs depending on the mode of initiation? Understanding how context-specific inputs are transformed into action within striatal circuits is essential for revealing the mechanisms of flexible motor control. At one end of the spectrum, neurons in the dorsal striatum may form entirely distinct populations that separately encode cues and actions, while at the other, the same neurons may exhibit overlapping activity that integrates both types of information, potentially along different population activity dimensions.

To address this question, we designed a task in which mice performed a simple, well-defined movement either spontaneously or in response to an external cue, enabling direct comparison of internally generated and stimulus-driven actions. By holding motor output constant, we isolated striatal activity differences that reflect initiation mode rather than movement kinematics, revealing how the striatum contributes to self-initiated versus cue-guided behavior. Two-photon calcium imaging in the DLS showed that, through unsupervised hierarchical clustering, neurons segregated into subpopulations tuned to the cue, movement, or postmovement epochs. One cluster was consistently active around movement regardless of initiation mode, identifying a shared action-timed ensemble. Despite this overlap, population dynamics diverged before movement onset, and trial type could be reliably decoded using support vector machines (SVMs). Within the same population, subspace analyses revealed orthogonal components encoding initiation context and movement execution. Both D1- and D2-SPNs participated in these subspaces—D1-SPNs showing elevated activation near cue onset and movement initiation and D2-SPNs exhibiting increased postaction activity across both trial types. These findings show that internal and external drivers of behavior converge onto shared action-encoding neurons, whose context-dependent dynamics define a flexible population code for action within basal ganglia circuits.

## RESULTS

### Alternating cue-evoked and self-paced lever task

**Fig. 1.**
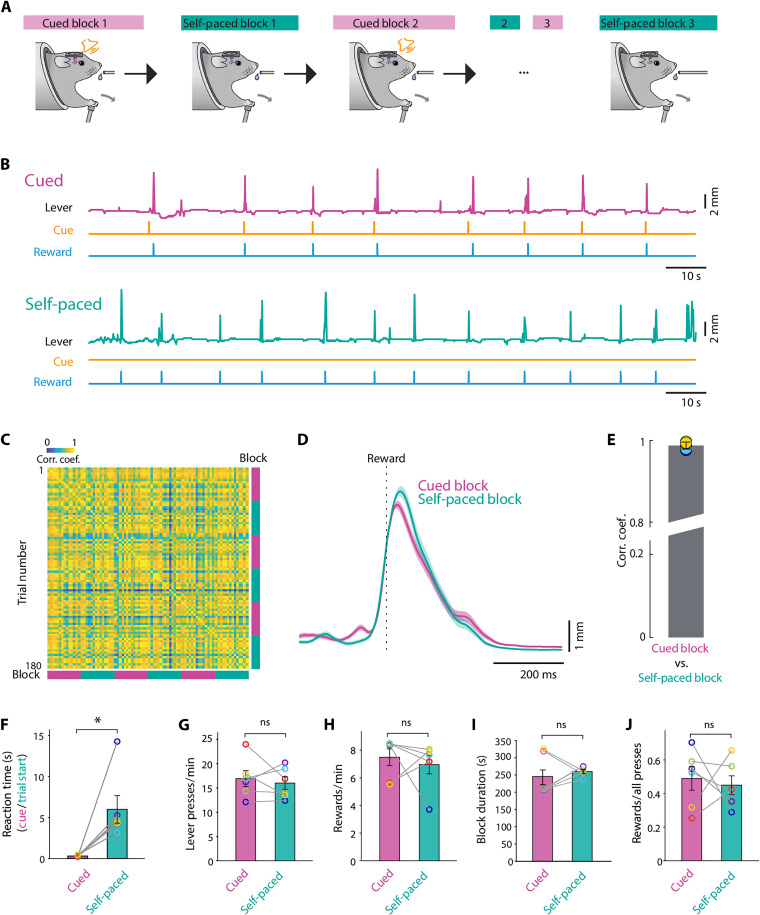
Switching task enables comparison of self-paced and cue-driven actions. (**A**) Schematic of the switching task depicting alternating block design within the same behavioral session. (**B**) Top: example of lever trace in a cued block (magenta). Bottom: example of a trace in a self-paced block (teal) from this same mouse. (**C**) Cross-correlation matrix of all rewarded lever push trajectories from an example mouse across all blocks during the test day. (**D**) Average lever press trajectories for self-paced and cued lever push trajectories for the same mouse as in (C). (**E**) The correlation coefficient between self-paced and cued lever push trajectories for all mice, plotted at mean ± SEM. (**F**) The reaction time after cue onset (cued blocks) and trial onset (self-paced blocks) was statistically significantly different in the two clock types (Wilcoxon signed rank test: *P < 0.05). (**G**) The lever push frequency (per minute) was not different between cued and self-paced blocks [Wilcoxon signed rank test: not significant (ns) *P* > 0.05]. (**H**) The rate of rewarded pushes (per minute) was not different between cued and self-paced blocks (Wilcoxon signed rank test: ns *P* > 0.05). (**I**) The block duration (in seconds) was not different between cued and self-paced blocks (Wilcoxon signed rank test: ns *P* > 0.05). (**J**) The fraction of lever pushes that led to reward was not different between cues and self-paced blocks (Wilcoxon signed rank test: ns *P* > 0.05). All bar plot data are represented as mean ± SEM. Each open circle represents an individual mouse.

We first developed a task in which mice learn to perform a self-paced and cue-evoked action within the same behavioral session, in different blocks. Food-deprived mice first learned to push a lever to receive a sucrose reward (fig. S1A). On subsequent training days, they learned to push the lever in response to an auditory cue (5 kHz, 100-ms pure tone). On the day of behavioral testing, mice performed these two behaviors in three sets of blocks, each of which began with a “cued block” followed by “self-paced block” (Fig. 1, A and B), with block transitions triggered after ∼30 rewards were obtained. In both block types, the rewarded lever trajectories were highly similar (Fig. 1, C to E). In addition, key parameters of lever press kinematics, including peak velocity, displacement, and press duration, did not differ between contexts (fig. S1, F to H). In cued blocks, mice pushed the lever in a manner that was time-locked with the cue, while in self-paced blocks, lever pushes occurred at variable times after the trial start which resulted in significantly shorter “reaction times” during the cued blocks (Fig. 1F, reaction time: cued = 0.30 s, self-paced = 6.0 s; Wilcoxon signed rank test, *P* = 0.03, *n* = 6). The rate of lever pushes was similar across block number within a block type (Fig. 1G, cued = 16.9 pushes/min; self-paced = 16.0 pushes/min; Wilcoxon signed rank test, *P* = 0.69, *n* = 6), as was the rate of rewarded lever pushes (Fig. 1H, cued = 7.49 rewards/min; self-paced = 7.0 rewards/min; Wilcoxon signed rank test, *P* = 0.84, *n* = 6). In addition, the total block duration was similar in both blocks (Fig. 1I, cued = 246 s; self-paced = 259 s; Wilcoxon signed rank test, *P* = 1, *n* = 6). This suggests that mice adopted a stable behavioral strategy rather than maximizing reward rate in the uncued condition, consistent with the task encouraging a consistent effort strategy across contexts. Last, the ratio of rewarded lever presses to all presses was not significantly different across the trial blocks in either block type (Fig. 1J, cued = 0.49; self-paced = 0.45; Wilcoxon signed rank test, *P* = 0.68, *n* = 6). This consistency in behavior across blocks indicates that performance was stable over time, allowing us to attribute differences between block types to task structure rather than changes in motivation or motor output. Inhibition of neural activity in the DLS with fluorescent muscimol significantly decreased task performance during both self-initiated behavioral sessions and cue-evoked sessions (fig. S1, F to H). This was in contrast to inhibition of the posterior or “tail” of the striatum in which fluorescent muscimol specifically had an effect on cue-evoked lever pressing (fig. S1, I to K). This finding highlights a functional dissociation along the anterior-posterior axis of the striatum, suggesting that the DLS is broadly necessary for task performance regardless of initiation context, whereas the posterior striatum plays a more selective role in mediating cue-driven actions.

### A shared movement-timed DLS ensemble across contexts

**Fig. 2.**
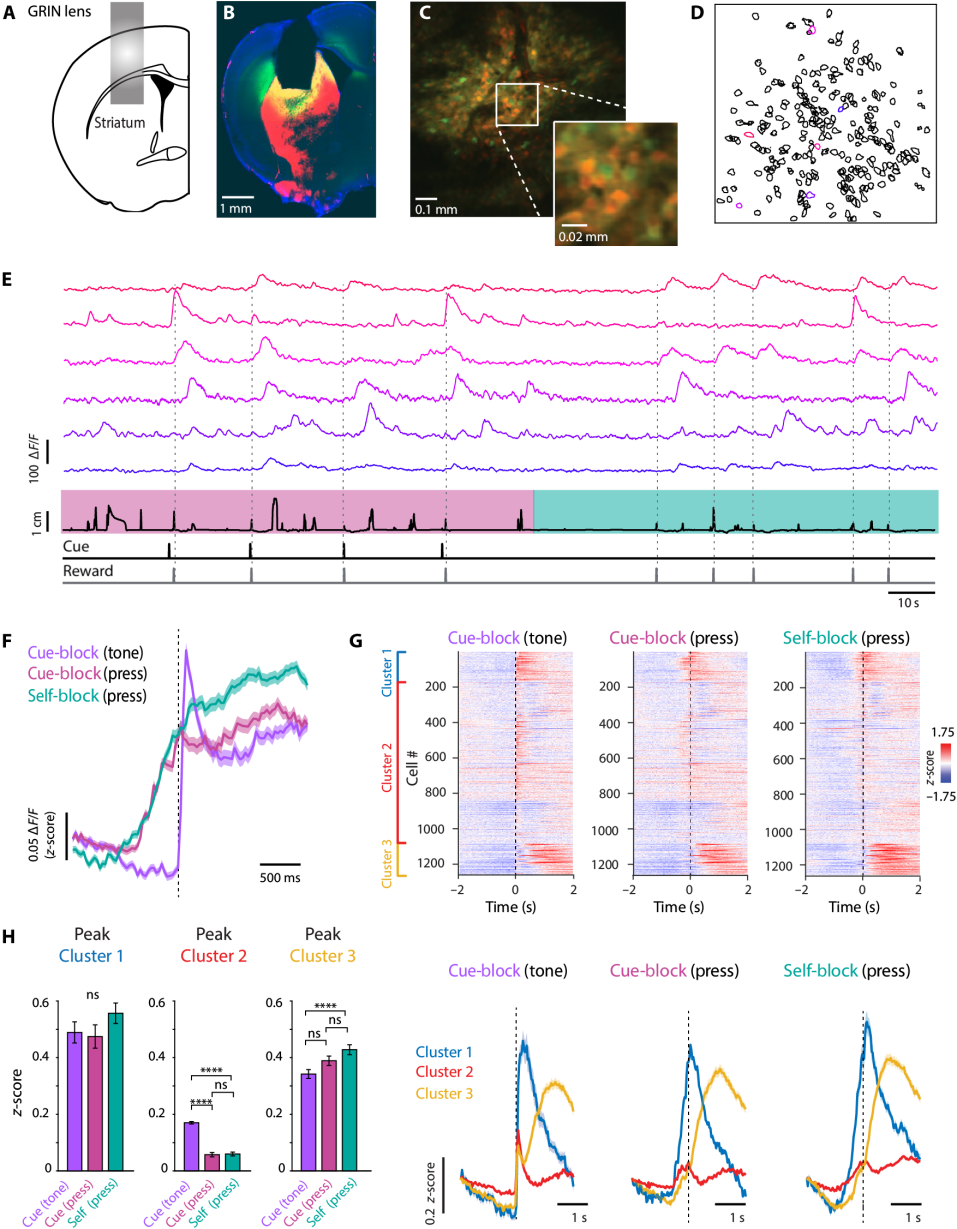
DLS population activity reveals shared motor representations across contexts. (**A**) Schematic of GRIN lens placement in the DLS. (**B**) Example histological verification of GRIN lens placement and GCaMP8m expression in a D2-tdTomato mouse. (**C**) Example field of view through the GRIN lens. (**D**) ROIs identified from a single session [same mouse as in (C)]. (**E**) Δ*F*/*F* traces for six example ROIs, with lever position and reward times shown below. Colors correspond to example cells shown in (D). (**F**) Average population activity of all SPNs aligned to cue onset (purple), rewarded presses in cued blocks (magenta), or rewarded presses in self-paced blocks (teal). (**G**) Top: Raster plots of SPN activity (each row, one neuron) aligned to cue onset (left), rewarded presses in cued blocks (middle), and rewarded presses in self-paced blocks (right). Neurons are ordered by hierarchical clustering based on activity during self-paced presses, defining three clusters [cluster 1, blue (*n* = 118); cluster 2, red (*n* = 868); cluster 3, yellow (*n* = 275)]. Bottom: Corresponding mean *z*-scored Δ*F*/*F* traces for each cluster. (**H**) Quantification of peak *z*-scored Δ*F*/*F* responses for each cluster. Cluster 1 showed similar responses across conditions [one-way analysis of variance (ANOVA): ns *P* > 0.05]. Cluster 2 exhibited larger responses to the cue than to press-aligned activity (one-way ANOVA: *P* < 1 × 10^−10^; Tukey-Kramer: ****P < 0.0001). Cluster 3 showed larger responses for self-paced press-aligned activity (one-way ANOVA: *P* = 0.002; Tukey-Kramer: ****P < 0.0001). Shaded areas and bar plots indicate mean ± SEM.

To investigate the neural activity underlying cue-evoked and self-paced lever pushing, we virally expressed the Ca^2+^ indicator GCaMP8m (*N* = 4) or GCaMP8f [*N* = 2; (*33*)] in the DLS (Fig. 2A). To distinguish between D1- and D2-SPNs, we performed experiments in mice genetically engineered to express tdTomato in D2-SPNs or D1-SPNs [generated by crossing D2-Cre (*N* = 3) or D1-Cre mice (*N* = 3); (*34*, *35*)] with Ai14 reporter mice [*N* = 3; (*36*)] (Fig. 2, B and C). This approach allowed Ca^2+^ signals arising from tdTomato-positive D1- or D2-SPNs to be distinguished from tdTomato-negative neurons. In the dorsal striatum, spiny projection neurons constitute ∼95% of the neuronal population, with interneurons representing only a small minority. To further minimize potential interneuron contamination, tdTomato-negative neurons exhibiting properties consistent with interneurons, such as elevated baseline activity, were excluded from subsequent analyses (*26*). Previous characterization of the EY217 (D1) and ER44 (D2) Gene Expression Nervous System Atlas (GENSAT) lines (*31*, *37*, *38*) shows that these drivers label ∼40 to 50% of DLS neurons with high pathway specificity, consistent with the expected proportions of D1- and D2-expressing spiny projection neurons. Imaging commenced on the first day they were required to alternate between cue-evoked and self-paced lever blocks, beginning with a cue block in all mice, as described above. Individual regions of interest (ROIs) were semiautomatically extracted using Suite 2P (*39*) and showed behaviorally relevant fluorescence changes (Fig. 2, D and E). We then took the grand trial average *z*-scored Δ*F*/*F* responses across all neurons in cue-evoked and self-paced blocks and aligned them to the time of the lever push (Fig. 2F, magenta and teal traces) and found them to be similar in 1 s leading up to the push action. Notably, when we aligned the trials in the cue-evoked blocks to the cue, we observed a sharp rise in average activity that coincided with the onset of the cue (Fig. 2F, purple trace).

Unsupervised clustering revealed that neurons (*n* = 1261) segregated into three functional ensembles, supported by silhouette analysis indicating optimal structure at *k* = 2 to 3 (fig. S2A). We selected *k* = 3, as this solution resolved distinct response profiles corresponding to cue-, movement-, and postaction-related activity one of which—cluster 1—was consistently active around lever pressing across both contexts. Notably, this shared movement-timed ensemble emerged spontaneously, underscoring that neurons encoding action are inherently shared across initiation modes. The activity of neurons belonging to each cluster can be visualized aligned either to the cue in cue-evoked blocks or to the press in both cue and self-paced blocks (Fig. 2G). Cluster 1 exhibited a peak response ∼200 ms after cue onset when aligned to it, which was also evident when aligned to the press (Fig. 2G, blue trace). There was no significant difference in peak amplitude of this cluster when aligned to the tone in cued blocks or to the press in either context [Fig. 2H, left; *z*-score responses: cued, tone aligned = 0.49 ± 0.04; cued, press aligned = 0.47 ± 0.04; self-paced, press aligned = 0.56 ± 0.04; *n* = 118; one-way analysis of variance (ANOVA), *P* = 0.27]. This indicates that neurons in cluster 1 are consistently engaged during lever pressing in both cue-evoked and self-initiated contexts, suggesting that a shared neural population participates in encoding across conditions.

Neurons in cluster 2 showed a grand average peak response well aligned with the cue, peaking ∼66 ms after tone onset. Their responses were significantly higher when aligned to the cue, indicating selective engagement by the tone (Fig. 2H, middle; *z*-score responses: cued, tone aligned = 0.17 ± 0.004; cued, press aligned = 0.06 ± 0.008; self, press aligned = 0.06 ± 0.007, *n* = 868; one-way ANOVA, *P* < 1 × 10^−10^). To dissociate cue-locked activity from movement-related responses, we separated trials into bins based on reaction time (e.g., 0 to 333 ms or 334 to 666 ms; fig. S2). In trials where the cue occurred within a defined time window before lever pressing, cluster 2 neurons exhibited responses tightly aligned to cue onset and occurring before movement initiation (fig. S2, E to H), consistent with this cluster primarily reflecting cue-driven activity. Last, cluster 3 neurons peaked ∼1 s after cue onset, consistent with licking and/or reward consumption. They showed the strongest responses in self-paced trials when aligned to the press (Fig. 2H, right; *z*-score responses: cued, tone aligned = 0.34 ± 0.02; cued, press aligned = 0.39 ± 0.02; self, press aligned = 0.43 ± 0.02; *n* = 275; one-way ANOVA, *P* = 0.0015). While *k* = 3 was selected on the basis of silhouette analysis and interpretability, clustering was used as a tool to identify functional response profiles—most notably a movement-aligned ensemble (cluster 1)—rather than to define discrete cell types. Together, these results show that DLS neurons segregate into three functional ensembles—one encoding movement regardless of initiation mode, one selectively engaged by external cues, and one linked to postaction reward–related activity—highlighting distinct but complementary roles in processing motor and contextual information.

### D1- and D2-SPNs exhibit distinct temporal dynamics around action execution and postaction periods

**Fig. 3.**
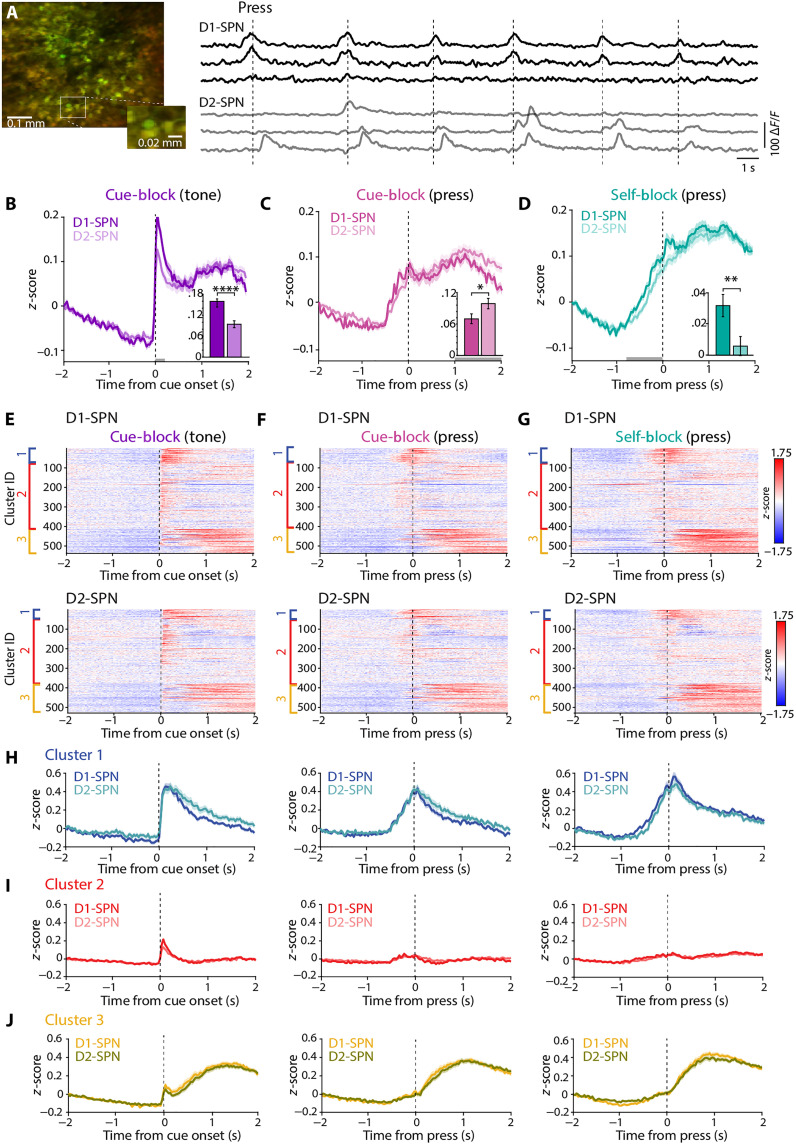
D1- and D2-SPN activity during the switching task. (**A**) Left: Two-photon image of a field of view showing GCaMP8m (green) and tdTomato (red) expression. Right: Example Δ*F*/*F* traces from putative D1-SPNs (black, top) and D2-SPNs (gray, bottom). (**B**) Average population activity of D1-SPNs (dark purple; *n* = 225 labeled, *n* = 313 putative) and D2-SPNs (light purple; *n* = 281 labeled, *n* = 248 putative) aligned to cue onset. Inset: Mean *z*-scored Δ*F*/*F* (0 to 200 ms after cue onset) was higher in D1-SPNs (Student’s *t* test: ****P < 0.0001). (**C**) Average population activity aligned to lever press in cued blocks (dark magenta, D1; light magenta, D2). Inset: Mean *z*-scored Δ*F*/*F* (1 to 2 s after press onset) was higher in D2-SPNs (Student’s *t* test: *P < 0.05). (**D**) Average population activity aligned to lever press in self-paced blocks (dark teal, D1; light teal, D2). Inset: Mean *z*-scored Δ*F*/*F* (−750 to 0 ms before press) was higher in D1-SPNs (Student’s *t* test: **P < 0.01). (**E** to **G**) Raster plots of D1-SPN (top) and D2-SPN (bottom) responses aligned to cue onset (E), press in cued blocks (F), and press in self-paced blocks (G). Neurons are ordered by cluster (cluster 1, blue; cluster 2, red; cluster 3, yellow). (**H** to **J**) Mean *z*-scored Δ*F*/*F* D1- and D2- SPN traces for clusters 1 to 3 aligned to cue onset (left), press in cued blocks (middle), and press in self-paced blocks (right). Shaded areas and bar plots indicate mean ± SEM.

We used our ability to identify neurons as D1-SPNs or D2-SPNs by their expression of tdTomato and analyzed our data after separating ROIs into putative D1-SPNs or D2-SPNs (Fig. 3A). We then plotted the grand average *z*-scored fluorescence changes for all neurons imaged across the six mice (*n* = 538 D1-SPNs, *n* = 529 D2-SPNs). When aligned to the cue, D1-SPNs showed significantly higher responses in the first 200 ms after the cue (Fig. 3B; mean *z*-score of 0 to 200 ms from cue onset: D1 = 0.16 ± 0.01, D2 = 0.09 ± 0.01, *P* = 2 × 10^−5^, Student’s *t* test). D2-SPNs showed significantly higher average responses in the period from 1 to 2 s after the cue onset (Fig. 3C; mean *z*-score of 1 to 2 s from cue onset: D1 = 0.07 ± 0.01, D2 = 0.10 ± 0.01, *P* = 0.03, Student’s *t* test). D1-SPNs also showed significantly higher activity in the time period immediately preceding the press in both cue-evoked and self-paced blocks (Fig. 3D; mean *z*-score  −750 to 0 ms from press onset in self-paced block: D1 = 0.03 ± 0.007, D2 = 0.006 ± 0.006, *P* = 0.006, Student’s *t* test). Example traces and quantitative comparisons of amplitude and event rate indicate similar event properties between GCaMP8f and GCaMP8m animals (fig. S3, A to D), suggesting that indicator kinetics did not drive the observed results. Notably, when restricting the analysis to definitively identified D1- and D2-SPNs (excluding putative classifications), these differences remained in the same direction and were evident at the population level (fig. S4, A to C), although they were more variable across individual animals (fig. S4, G to I).

Separating the previously clustered neurons into D1- and D2-SPNs revealed a similar proportion of each cluster across both cell types (fig. S5E). Cluster 1 neurons were active at cue onset and around the press in both trial types; within this cluster, D2-SPNs showed higher activation in the period of 1 to 2 s after the cue, before the next intertrial interval (Fig. 3H and fig. S5, A to C). Cluster 2 neurons were most strongly aligned to the cue, with D1-SPNs showing significantly higher responses in the 0- to 200-ms window after cue onset (Fig. 3I, left, and fig. S5D). Cluster 3 neurons peaked later, around reward and licking, and showed similar activation between D1- and D2-SPNs (Fig. 3J). These results reveal that differences between D1- and D2-SPNs are not homogeneous across the population but emerge in specific functional clusters—D1-SPNs are more engaged during early cue processing and movement preparation in certain ensembles, whereas D2-SPNs show enhanced postaction activation in others—indicating that each pathway contributes to initiation and evaluation processes in a cluster- and context-dependent manner.

### Neural dynamics predict cue-evoked versus self-paced actions

**Fig. 4.**
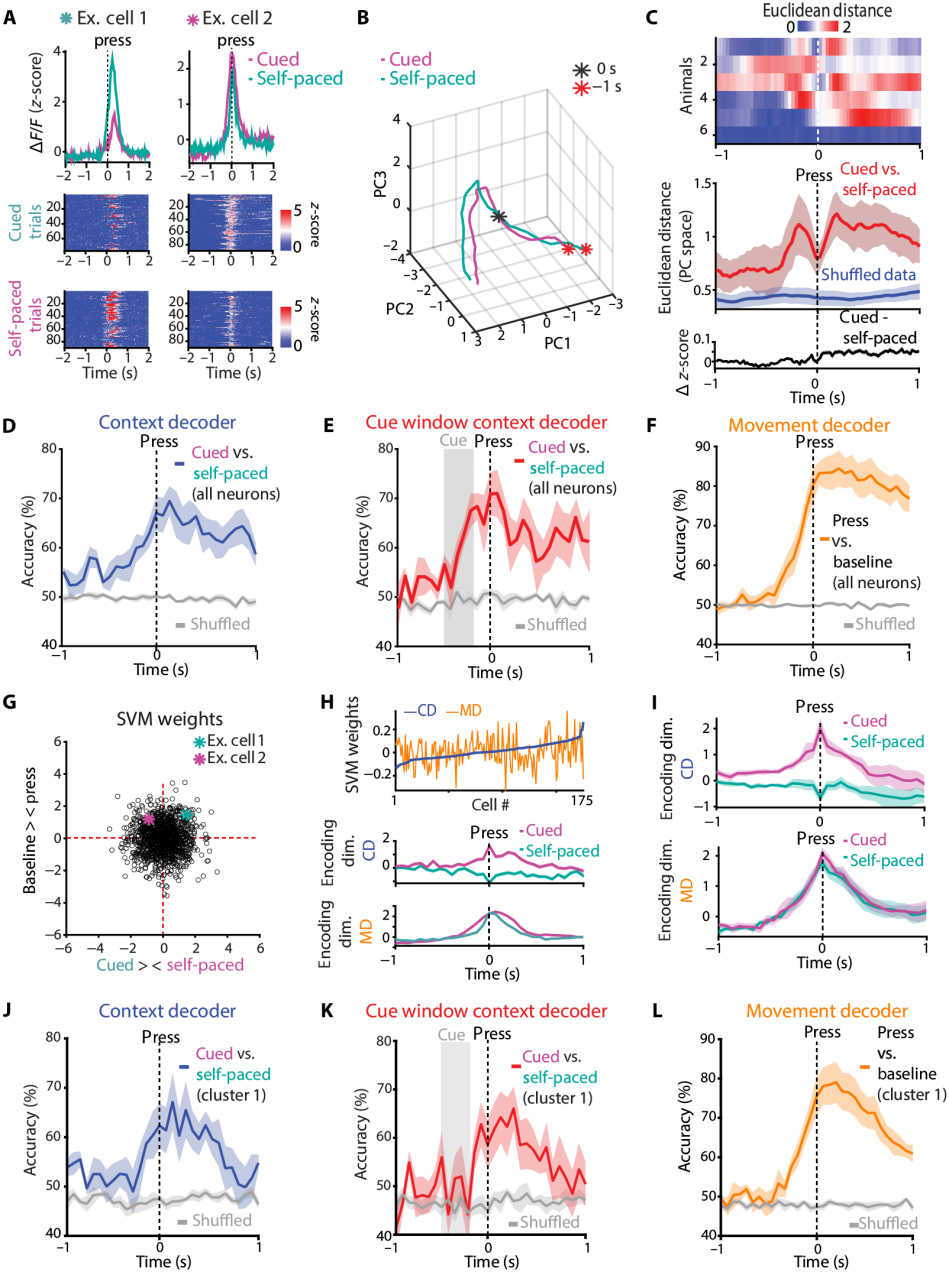
Shared and distinct neural dimensions underlying self-paced and cue-evoked actions. (**A**) Example neurons showing responses to both cue-evoked (magenta) and self-paced (teal) lever presses, with differing magnitudes. Top: grand average *z*-scored responses; bottom: raster plots for cued and self-paced trials. (**B**) PCA of population activity in an example mouse showing neural trajectories for cue-evoked (magenta) and self-paced (teal) trials. (**C**) Top: Euclidean distance between neural trajectories for cue-evoked and self-paced trials for individual mice. Middle: mean Euclidean distance across mice (red) compared to shuffled data (blue), showing divergence during preparation and reward and convergence at lever press. Bottom: subtraction of grand-average ΔF/F lever-aligned traces, indicating that trajectory differences are not explained by mean activity differences. (**D**) SVM context decoder predicts trial type above chance; gray indicates shuffled data. (**E**) Cue window context decoder accuracy rises above chance during the cue period (−495 to −165 ms before press) for matched-latency trials; gray indicates shuffled data. (**F**) Movement decoder distinguishes lever press from baseline above chance before movement onset, indicating a shared preparatory dimension; gray indicates shuffled data. (**G**) Distribution of SVM weights across neurons; example neurons from (A) are highlighted. (**H**) Top: decoder weights for context decoder (CD, blue) ordered by weights and movement decoder (MD, orange) showing that neurons strongly predictive of context prediction do not systematically contribute to movement. This indicates that movement- and context-related signals are distributed across partially independent neural dimensions rather than a single shared axis. Middle: Projection onto the context dimension separates cued and self-paced trials. Bottom: Projection onto the movement dimension shows similar modulation across contexts. (**I**) Same as (H), middle and bottom, for all neurons pooled across mice. (**J** to **L**) Same as (D) to (F), restricted to cluster 1 neurons. Shaded areas indicate ±SEM.

The shared movement-timed neurons (cluster 1) provided a foundation for testing whether population dynamics—rather than cell identity—differentiate internally and externally initiated actions. To do so, we examined features such as baseline activation levels, the temporal dynamics of activity buildup, and used a SVM classifier to assess whether patterns of population activity could reliably predict trial type. These approaches allowed us to explore whether the mode of initiation is reflected not in the identity of responsive neurons but in the structure or timing of their activity. While many cells were responsive during both cued and self-paced lever presses, only a small fraction exhibited differences in response amplitude across contexts, and these effects were heterogeneous across neurons (Fig. 4A and fig. S6A). Principal components analysis (PCA) of the population activity in an example mouse revealed that, although neural trajectories began in a similar state across conditions, they diverged during the preparatory period and then converged again at the time of reward delivery (Fig. 4B). This suggests that convergence at reward could reflect a shared outcome-related signal across initiation modes. Across all mice, the Euclidean distance between neural trajectories peaked several hundred milliseconds before the lever press, indicating a growing divergence in preparatory activity between cued and self-paced trials. This distance then dipped at the time of the press, reflecting transient convergence in neural dynamics associated with movement execution. This was not a trivial consequence of differences in the overall averages, as subtraction of the grand average cue-evoked and self-evoked traces did not show peaks either leading up to or following the press (Fig. 4C).

We next asked whether patterns of population activity contained information about the mode of initiation. To test this, we trained an SVM classifier on 90% of the data and evaluated its performance on the remaining 10%, asking whether it could predict whether a given lever press was cue-evoked or self-initiated. The classifier (“context decoder”) performed above chance, with accuracy peaking at and just after movement onset (Fig. 4D), indicating that population dynamics around the time of execution contain features that distinguish initiation mode. When restricted to cued trials with similar reaction times (“cue window context decoder”), classification accuracy rose above chance even during the cue period, showing that predictive signals emerge before movement and likely reflect context-specific preparatory activity (Fig. 4E). Decoding performance remained slightly above chance during intertrial periods, consistent with the presence of residual population activity that may weakly encode contextual information outside the immediate movement epoch.

In parallel, we trained an SVM to discriminate lever presses—regardless of initiation context—from baseline (“movement decoder”). This classifier also performed above chance, beginning several hundred milliseconds before the press, revealing a shared, context-invariant preparatory dimension that generalized across action types (Fig. 4F). Thus, decoding analyses uncovered two complementary components of striatal activity: one encoding the context in which an action is generated and another capturing a common motor execution signal that is conserved across contexts.

Examining classifier weights showed that discriminating information was distributed broadly across the population rather than concentrated in a small subset of cells (Fig. 4G). To further assess how information was distributed across neurons, we performed an additional analysis in which the SVM was trained sequentially by adding one neuron at a time, generating curves showing classifier performance as a function of population size for each animal separately. This analysis revealed a gradual increase in decoding accuracy as additional neurons were included, indicating that context-discriminating information is distributed across the population rather than driven by a small number of cells. Notably, decoding accuracy exceeded chance levels in all animals when the full recorded population was included (fig. S6C). Specifically, when neurons were ordered by their weights in the movement decoder, the corresponding weights in the context decoder were distributed independently, indicating that neurons most predictive of movement were not preferentially informative about context. Consistent with this, projections of population activity onto the context-predictive dimension revealed clear separation between cued and self-paced trials, whereas projections onto the movement-predictive dimension showed similar modulation for both contexts. This dissociation was evident both at the level of individual animals and in the pooled population (Fig. 4, H and I). Together, these results demonstrate that context-invariant movement signals and context-specific signals are encoded along separable axes of population activity, rather than arising from a single shared dimension.

To determine whether different functional ensembles contributed differently to context decoding, we trained the SVM classifier separately on neurons belonging to each cluster. This analysis revealed that cluster 1 neurons—those with robust action-related responses shared across cue-evoked and self-paced trials—were unexpectedly effective at distinguishing initiation mode (Fig. 4J). Despite showing highly similar press-aligned activity in both trial types, cluster 1 neurons supported above-chance classification of cue versus self-paced presses, performing as well as or better than cluster 2 (fig. S7, C to G), which contained cue-selective neurons. Decoding accuracy for trial type using cluster 1 activity remained at chance during the prepress cue period (Fig. 4K). In contrast, cluster 1 activity reliably predicted lever pressing versus baseline (Fig. 4L), consistent with its role in shared motor preparation rather than encoding the sensory cue. This suggests that neurons that generalize across contexts at the single-cell level can nonetheless contribute to context-specific coding at the population level, highlighting how separable dimensions of activity within overlapping ensembles enable the striatum to flexibly represent both action execution and initiation mode.

Last, when we performed PCA and trained SVM classifiers separately on D1- and D2-SPNs, both populations supported above-chance classification of trial type with similar accuracy. This indicates that context differentiation arises from population-level organization rather than cell type segregation, despite distinct temporal profiles of D1- and D2-SPNs (fig. S8).

## DISCUSSION

Our study reveals that the DLS encodes both externally triggered and internally generated actions through overlapping but distinguishable neural dynamics. Using a task that held motor output constant, we identified through unsupervised clustering a single population—cluster 1—that consistently aligned with movement, regardless of initiation mode. We did not preselect these neurons; rather, they emerged from unsupervised hierarchical clustering as the only action-timed ensemble, underscoring that neurons encoding action are inherently shared across contexts. Critically, this indicates that internally generated and externally triggered actions are not implemented by segregated neural populations but instead arise from activity within a shared ensemble. Yet despite this overlap, the population dynamics within these shared neurons diverged before movement, reflecting distinct preparatory trajectories for cue-evoked versus self-initiated actions. This finding is consistent with recent work showing that dopamine dynamics in the DLS track the timing and initiation of upcoming actions (*40*) and indicates that convergence between internal and external drivers does not occur only at the level of motor output (e.g., muscles or descending commands) but rather within the striatal population space itself. Thus, context is not encoded by recruiting distinct circuits but by shaping trajectories within a shared population.

The striatum thus forms a high-level “action space” where contextual information is integrated and transformed into a common execution signal—a context-generalizable code for action. In this view, flexibility in initiation arises not from switching between specialized circuits but from dynamic reconfiguration within shared ensembles that can encode both context and action along orthogonal dimensions. This framework shifts the interpretation of striatal function from selecting between competing pathways to organizing behavior through population-level dynamics within a common representational space. This organization echoes emerging principles of population-level computation described in cortical circuits, where overlapping neurons support diverse functions through mixed selectivity and structured neural subspaces (*41*–*43*). In contrast to motor cortex, where preparatory activity converges across self-initiated and cue-driven movements (*6*), the striatum differentiates initiation context within shared ensembles, transforming convergent cortical plans into context-specific population dynamics.

Our ability to distinguish D1- and D2-SPNs further revealed distinct contributions of these two striatal subpopulations to context encoding. D1-SPNs were preferentially engaged during early cue processing and just before movement onset, consistent with a facilitative role during externally triggered actions. Consistent with our previous work, D1-SPNs exhibited stronger responses to the auditory cue than D2-SPNs (*31*); however, unlike that study, which examined sensory responses in the tail of the striatum, these results are observed in the DLS during a learned instrumental action. By contrast, D2-SPNs exhibited greater activity following lever presses, particularly in the postcue and intertrial intervals, suggesting a role in suppressing or regulating ongoing behavior (*44*, *45*). These results add to a growing body of work suggesting that direct and indirect pathway neurons are coengaged during behavior, indicating that both contribute to shaping action initiation and evaluation in a context- and cluster-dependent manner.

The simplicity of our behavioral paradigm is both a limitation and a strength. The one-dimensional lever press does not capture the richness of naturalistic motor sequences, but its stereotypy allows precise control over kinematics, isolating neural differences that reflect initiation context rather than movement variability. This design was essential for revealing that shared striatal populations flexibly encode both self-initiated and cue-triggered actions. Extending this framework to more complex, multidimensional behaviors will help determine whether the context- and action-related population subspaces identified here generalize across the broader motor repertoire.

## MATERIALS AND METHODS

### Animals

All experiments were carried out with 8- to 16-week-old male and female mice > 15 g in accordance with National Institutes of Health guidelines and protocols approved by the NYU Langone Health (NYULH) Institutional Animal Care and Use Committee (protocol #PROTO201900059, PROTO201900067). No effects of sex are reported. Mice were bred in-house on a C57Bl/6J background and housed in a reverse light/dark cycle (light 11 p.m. to 11 a.m.), at a temperature of 22° ± 2°C with food available ad libitum. Mice were group housed with littermates of the same sex during experimental procedures. The following strains used in this proposal were sourced from the Jackson Laboratory: Ai14: B6.Cg-*Gt(ROSA)26Sor^tm14(CAG-tdTomato)Hze^*/J (JAX:007914). The following bacterial artificial chromosome (BAC) transgenic mouse strains used in this paper were sourced from the Mutant Mouse Regional Resource Centers (MMRRC): D2-Cre: B6.FVB(Cg)-Tg(Drd2-cre)ER44Gsat/Mmucd (MMRRC_032108-UCD); D1-Cre: Tg(Drd1-cre)EY217Gsat/Mmucd (MMRRC_030778-UCD).

All experiments were carried out with 8- to 16-week-old male and female mice in accordance with protocols approved by the NYULH Institutional Animal Care and Use Committee (protocol #PROTO201900059). D2-Cre BAC transgenic mice were obtained from GENSAT (founder lines EY217 for D1-Cre and ER44 for D2-Cre) and purchased through the MMRRC. These mice were crossed with Lox-Stop-Lox-tdTomato mice (Ai14, JAX:007914). The mice were housed in a reverse light/dark cycle (light 11 p.m. to 11 a.m.), at a temperature of 22° ± 2°C with food restriction beginning 24 to 48 hours before the commencement of behavioral training.

### General surgical procedures

Mice were anesthetized with isoflurane (2.5 to 3%, plus oxygen at 1 to 1.5 liters/min) and then placed in a stereotaxic holder (Kopf Instruments). Meloxicam (5 mg/kg) was administered subcutaneously for analgesia before the onset of surgery. The scalp was shaved and disinfected using 70% alcohol followed by iodine, and intradermal bupivacaine (2 mg/kg) was also provided for local anesthesia. The mouse’s body temperature was maintained throughout surgery at 37°C using an animal temperature controller [Future Health Concepts (FHC) DC Temperature Controller]. After surgery, mice were allowed to recover until ambulatory in the home cage on a heating pad. Additional analgesia was administered with ibuprofen (100 mg/ml) in the drinking water for 3 days postsurgery.

### Chronic GRIN lens implantation

Animals were unilaterally injected with 500 nl of AAV.CamKIIa.jGCaMP8m.WPRE (Addgene ref #176751-AAV9, lot #v159649, 2.7 × 10^13^ vg/ml) or AAV.syn.jGCaMP8f.WPRE [Addgene ref #162376-AAV9, lot #v136270, 2.3 × 10^13^ genome copies (GC)/ml]. Recordings were obtained from three D1-Cre (EY217)/Ai14 mice, all expressing jGCaMP8m, and three D2-Cre/Ai14 mice, of which one expressed jGCaMP8m and two expressed jGCaMP8f. Injections were performed in the DLS [coordinates: anterior posterior (AP) of +0.5 mm mediolateral (ML) of −2.2 mm, and dorsoventral (DV) of −2.6 mm from bregma]. Tissue was then aspirated at a circumference of ∼1 mm to a depth ∼200 μm above the center of the injection site. A 1.0-mm gradient index (GRIN) lens [length: ∼4.38 mm, working distance: image side: 0.1 mm in air, object side: 0.25 mm in water, design wavelength: 860 nm, numerical aperture (NA) 0.5, noncoated; catalog no. 1050-006242, Inscopix Inc.] was then lowered ∼2 mm in depth, stopping ∼200 to 400 μm above the center of the injection site. The lens was secured in place with glue, and a headpost was implanted with C&B Metabond dental cement (Parkell) around the lens.

### Behavioral training and monitoring

Mice were subjected to a food restriction schedule and were maintained at 85% of their starting weight. We implemented a custom head restrained lever-based training paradigm where mice push the lever spontaneously (“self-initiated” behavior) or push the lever in response to an auditory cue (“cued” behavior) to collect 5 μl of a 10% sucrose solution reward. We used an adapted custom designed lever (*46*), which was mounted onto a rotary encoder (US Digital) 4 cm from the lever handle. A magnet (CMS magnets) was mounted to the bottom of the lever and positioned 1.5 cm above a static magnet that established the resting position of the lever and provided a way to alter the movement resistance. The lever handle was positioned adjacent to a cup [copyright IR CU21353, three-dimensional (3D) printed using a MakerBot Replicator+ 3D Printer] to hold mice below two plate clamps for head fixation.

The task required the mice to push the lever across a threshold of 3 mm of displacement to receive a reward. In self-initiated blocks, mice could get a reward when they spontaneously pushed the lever past the reward threshold. However, an “intertrial interval” was superimposed such that after a reward was given, 2 to 3 s needed to pass before another reward could be delivered. In addition, to discourage the mice from continuously pushing the lever, mice had to hold the lever at the home position (“quiet period”) for at least 1 to 2 s in before a rewarded lever press. In cued blocks, the intertrial interval was 4 to 8 s, after which, if the mice held the lever in the home position for a period of 1.5 to 2.5 s, a “go” cue (5-kHz pure tone) would be delivered. If mice pushed the lever past the reward threshold within 2 s after this cue, then they would receive the reward. Training consisted in three stages. First, mice were rewarded for spontaneous lever pushes for 3 days of training. During this phase, the intertrial interval (ITI) was held at 2 to 3 s, and the mice had to hold the lever still for at least 1 to 2 s between trials. Next, mice were trained to push the lever in response to the auditory cue, achieving a >80% “hit” rate over a period of 5 to 7 training days. Last, on the day of the recordings, mice alternated between cued and self-paced blocks, with each block lasting for a total of ∼30 rewards.

### Two-photon calcium imaging

All imaging experiments were conducted on an Ultima 2Pplus two-photon laser-scanning microscope (Bruker Inc.). The system was configured with 8-kHz resonant-galvo-galvo laser scanning mirrors, and imaging frames of 512 × 512 pixels (corresponding to an area of 1000 μm by 1000 μm) were acquired at 30 fps. The system was equipped with two-channel fluorescence detection with amplified noncooled gallium arsenide phosphide (GaAsP) photomultiplier tubes (PMTs). Emitted fluorescence was first directed to the PMTs and then split into “green” and “red” channels by a 565-nm sharp edge long-pass dichroic mirror. The green channel and the red channel were subsequently filtered by a 525-nm/39-nm bandpass filter and 593-nm/40-nm bandpass filter, respectively, before detection in the PMTs. The microscope was controlled via Prairie View version 5.6.

Imaging was performed via a Nikon 16×, 0.8 NA water immersion objective placed over the implanted GRIN lens. A Coherent Chameleon Vision II tunable titanium-sapphire laser tuned to 920 nm with 75-fs pulses at a repetition rate of 80 MHz was used. Dispersion correction was adjusted to maximize fluorescent brightness as recorded under the objective. The imaging power was modulated through a Conoptics 350-105 Pockels Cell driven by a Conoptics 302 RM Amplifier.

Behavioral data including lever presses and video data were recorded using a Hardware for Accelerating Research and Production project (HARP) Behavior board (https://open-ephys.org/harp/oeps-1216) and custom workflows using the BONSAI (https://bonsai-rx.org/) software package. Behavioral and imaging data were synchronized by sending Transistor-Transistor Logic (TTL) pulses from the two-photon microscope to the HARP behavior board every time a new two-photon frame was recorded. These timestamps could then be used to align and down-sample behavioral data to match imaging data using custom MATLAB scripts.

### Muscimol infusions

Adult mice (C57BL/6J, male or female, aged 8 to 12 weeks) were implanted with unilateral guide cannulae targeting the dorsal striatum. Mice were anesthetized and prepared for surgery as described above. A small craniotomy was made at the target coordinate for the DLS (anteroposterior: +0.5 mm, mediolateral: ±2.2 mm from bregma) or the posterior tail striatum (anteroposterior: −1.6 mm, mediolateral: ±3.1 mm from bregma), and a 26-gauge stainless steel guide cannula (Plastics One) was lowered to 0.5 mm above the final infusion site (dorsoventral: −2.15 mm for DLS, −2.75 for posterior striatum from the skull surface). The cannula was secured to the skull using dental cement (C&B Metabond). A dummy cannula was inserted to prevent occlusion. Mice were allowed to recover for at least 7 days before beginning behavioral or infusion experiments. Postoperative analgesia (meloxicam, 5 mg/kg, sc) was administered once daily for 3 days.

For acute inactivation of striatal activity, fluorescent muscimol [Muscimol, BODIPY TMR-X Conjugate; 0.5 mg/ml in sterile phosphate-buffered saline (PBS)] was infused unilaterally through an internal injector extending 0.5 mm beyond the guide cannula tip. Mice were gently head-fixed for infusion procedures.

A total volume of 350 nl fluorescent muscimol was delivered at a rate of 0.1 μl/min using a microsyringe pump (Hamilton). After infusion, the injector was left in place for an additional 1 min to allow diffusion and minimize backflow. The injector was then removed and replaced with the dummy cannula. Control animals received equal volume infusions of sterile PBS.

Behavioral testing commenced 30 min after infusion to ensure effective onset of muscimol action. The location of the infusion site was later verified histologically.

### Immunohistochemistry

Mice were deeply anesthetized with isoflurane (5% for 5 to 8 min, inhaled) and perfused transcardially with 4% paraformaldehyde (PFA) in 0.1 M PBS. Brains were removed and fixed in 4% PFA for a maximum of 24 hours in the same solution, which was then replaced by a 0.1 M PBS solution, and 100-μm coronal slices were cut (vibratome, Leica VT1000S). The slices were subsequently mounted with 4′,6-diamidino-2-phenylindole mounting media (Southern Biotech). Images were obtained with an Olympus VS2000 slide scanner.

### Quantification and statistical analysis

#### Calcium imaging data preprocessing

Calcium imaging data were motion corrected using nonrigid motion correction, cells were segmented, and raw fluorescence traces were extracted using Suite2p (0.10.0) (*5*). Automatically classified cells were further manually curated using the Suite2p graphical user interface (GUI), and occasional ROIs with clearly nonphysiological morphological features (order of magnitude to large/small), no transients or clearly nonphysiological activity patterns (highly regular or square shaped fluctuations), were excluded.

Using custom a MATLAB code, raw fluorescence traces were neuropil-corrected by subtracting 0.7 times the neuropil signal from the raw cellular fluorescence. For each neuron, the fluorescence signal was then detrended to compute Δ*F*/*F*. This was done by estimating the baseline activity for each cell by applying Gaussian smoothing followed by a 50th percentile filter within nonoverlapping 60-s windows across the full recording. The resulting baseline trace was subtracted from the raw signal and divided by the same baseline to yield a Δ*F*/*F* signal, expressed as a percentage. Any traces with negative baseline values were excluded. The number of neurons excluded as putative interneurons was similar across animals (mouse 1: *n* = 3; mouse 2: *n* = 2; mouse 3: *n* = 5; mouse 4: *n* = 4; mouse 5: *n* = 3; mouse 6: *n* = 1).

After baseline correction, each Δ*F*/*F* trace was *z*-scored across the entire imaging session. These *z*-scored traces were then used for all subsequent analyses, including trial alignment, averaging, and dimensionality reduction.

#### Identification of D1- and D2-SPNs

To classify individual neurons as either direct pathway spiny projection neurons (D1-SPNs) or indirect pathway spiny projection neurons (D2-SPNs), we used a custom MATLAB GUI to manually inspect each ROI. Classification was based on the spatial correspondence between GCaMP-expressing cell bodies and tdTomato fluorescence. The experimenter was blinded to the experimental condition during this process. In EY217xAi14 mice, ROIs were classified as putative D1-SPNs if a tdTomato-positive signal spatially overlapped with the GCaMP signal and the shape of the labeled soma was clearly distinguishable from the background. In contrast, ROIs lacking a tdTomato signal, or showing a nonoverlapping tdTomato shape, were classified as putative D2-SPNs. For D2-Cre x Ai14 mice, the criteria were reversed: overlapping GCaMP and tdTomato signals indicated putative D2-SPNs, while tdTomato-negative or mismatched cells were categorized as putative D1-SPNs. Although GCaMP emission can extend weakly into longer wavelengths, tdTomato-positive neurons were readily distinguishable due to their substantially brighter somatic fluorescence in the red channel. To avoid misclassification due to spectral bleed-through, cells were designated tdTomato-positive only when clear somatic red fluorescence above local background was present and exceeded the weak diffuse red channel signal occasionally observed from GCaMP emission. Across all EY217 × Ai14 mice, we analyzed 225 tdTomato-positive D1-SPNs and 248 tdTomato-negative putative D2-SPNs. Across D2-Cre × Ai14 mice, we analyzed 281 tdTomato-positive D2-SPNs and 313 tdTomato-negative putative D1-SPNs. ROIs that could not be confidently classified on the basis of the criteria described above were excluded from cell type–specific analyses. The proportion of neurons excluded from D1/D2 classification varied across animals (range: 0 to 35%; mouse 1 = 9%; mouse 2 = 0%; mouse 3 = 1.7%; mouse 4 = 35%; mouse 5 = 19.5%; mouse 6 = 11%), reflecting differences in labeling clarity and imaging quality across the field of view, particularly for cells located near the periphery of the GRIN lens where classification was less reliable.

#### Population activity analyses

To compute peri-event trial-averaged activity, the neural activity of individual neurons was aligned to task-relevant events (i.e., cue onset or lever threshold crossing) and averaged across trials. For each neuron, calcium traces were first *z*-scored and then segmented into peri-event windows (−5 to +5 s, sampled at 30 Hz) around each event. Trials contaminated by nonphysiologically large artifacts were excluded using amplitude thresholds. For each event type (cued, uncued, or cue only), the trial-averaged activity of each neuron was computed by taking the mean trace across all valid trials. To compare average population activity across task blocks, the trial-averaged activity of all cells from all animals was pooled and averaged.

#### Hierarchical clustering

To identify functional subpopulations of neurons, we performed hierarchical clustering on the trial-averaged activity of all recorded neurons (*n* = 1271) aligned to the cue in cue-evoked trials. Each neuron’s response was defined as its *z*-scored calcium trace in a time window spanning 4 s around the cue (−2 to +2 s). We computed the pairwise Euclidean distance between these activity traces and applied the Ward’s method for agglomerative hierarchical clustering. To guide cluster selection, we computed the mean silhouette score across a range of cluster numbers (*k* = 2 to 6), which indicated that *k* = 2 to 3 provided the strongest structure. We therefore selected *k* = 3, as this solution yielded separable clusters corresponding to cue-, movement-, and postaction-related activity patterns. Neurons were grouped by cutting the dendrogram at *k* = 3, and the resulting cluster assignments were used to sort neurons for subsequent visualization across task conditions.

### PCA and population rate vector analysis

For population trajectory analysis, we categorized trials into self-paced (uncued) and externally triggered (cued) actions and computed the trial-averaged trajectory in the space of the first three principal components for each condition. Euclidean distance between the trajectories was calculated at each time point to quantify their divergence over time. As a control, we repeated the analysis 100 times with randomly shuffled trial labels to generate a null distribution of distances and compute the mean and SE of the shuffled results.

### Support vector machine

To classify cue-based versus self-initiated lever presses using neuronal activity, we trained a linear SVM classifier on calcium imaging data from individual animal sessions. We trained a linear SVM to classify trial type using population activity, where each sample corresponded to a trial (or time bin) and each feature corresponded to the activity of an individual neuron. Equal numbers of trials from each condition were randomly subsampled, and for each trial, fluorescence signals were aligned to behavioral events and segmented into 66-ms bins (two imaging frames per bin) across a 4-s peri-event window. At each time bin, the SVM classifier was trained on the population activity vector composed of the binned activity of individual neurons. Thus, each neuron contributed a separate feature to the classifier, allowing the SVM to leverage population activity rather than averaged signals. Classification was performed using 20-fold cross-validation. This procedure was repeated across all time bins to obtain a time-resolved decoding performance curve, with the resulting accuracies averaged across folds and sessions for further analysis. To estimate chance-level performance, we randomly shuffled the condition labels (cue-evoked versus self-initiated) across trials while keeping the underlying calcium activity unchanged. We then retrained the SVM using the same cross-validation procedure, repeating this process multiple times to generate a null distribution of decoding accuracies for comparison with the real data.

### Statistical analysis

Data are presented as mean ± SEM. Statistical analyses were performed using a MATLAB custom code. For unpaired datasets, two-tailed Student’s *t* tests (for normally distributed datasets) or Mann-Whitney tests (for nonnormally distributed datasets) were used. For paired datasets, two-tailed Student’s paired *t* test or Wilcoxon signed-rank test (for nonnormally distributed datasets) was employed. For multiple comparisons, we used two-way ANOVA followed by Sidak’s test. Values of *P* < 0.05 were considered statistically significant. *P* values are reported as follows: *P < 0.05, **P < 0.01, ***P < 0.001, and ****P < 0.0001. In all plots, unless otherwise noted, errors are plotted as ±SEM.
