# Supplementary material for: Shared striatal neurons exhibit context-specific dynamics for internally and externally driven actions
Source: Sci Adv. 2026 Jul 29;12(31):eaed9386. doi: 10.1126/sciadv.aed9386 (PMC13418543; doi:10.1126/sciadv.aed9386)
Supplement: Supplementary file 1 — Figs. S1 to S8 [file sciadv.aed9386_sm.pdf]

Science Advances
AAAS

Supplementary Materials for

Shared striatal neurons exhibit context-specific dynamics for internally and externally driven actions

Jan L. Klee *et al.*

Corresponding author: Tanya Sippy, tanya.sippy@nyulangone.org

Sci. Adv. **12**, eaed9386 (2026)
DOI: 10.1126/sciadv.aed9386

This PDF file includes:

Figs. S1 to S8

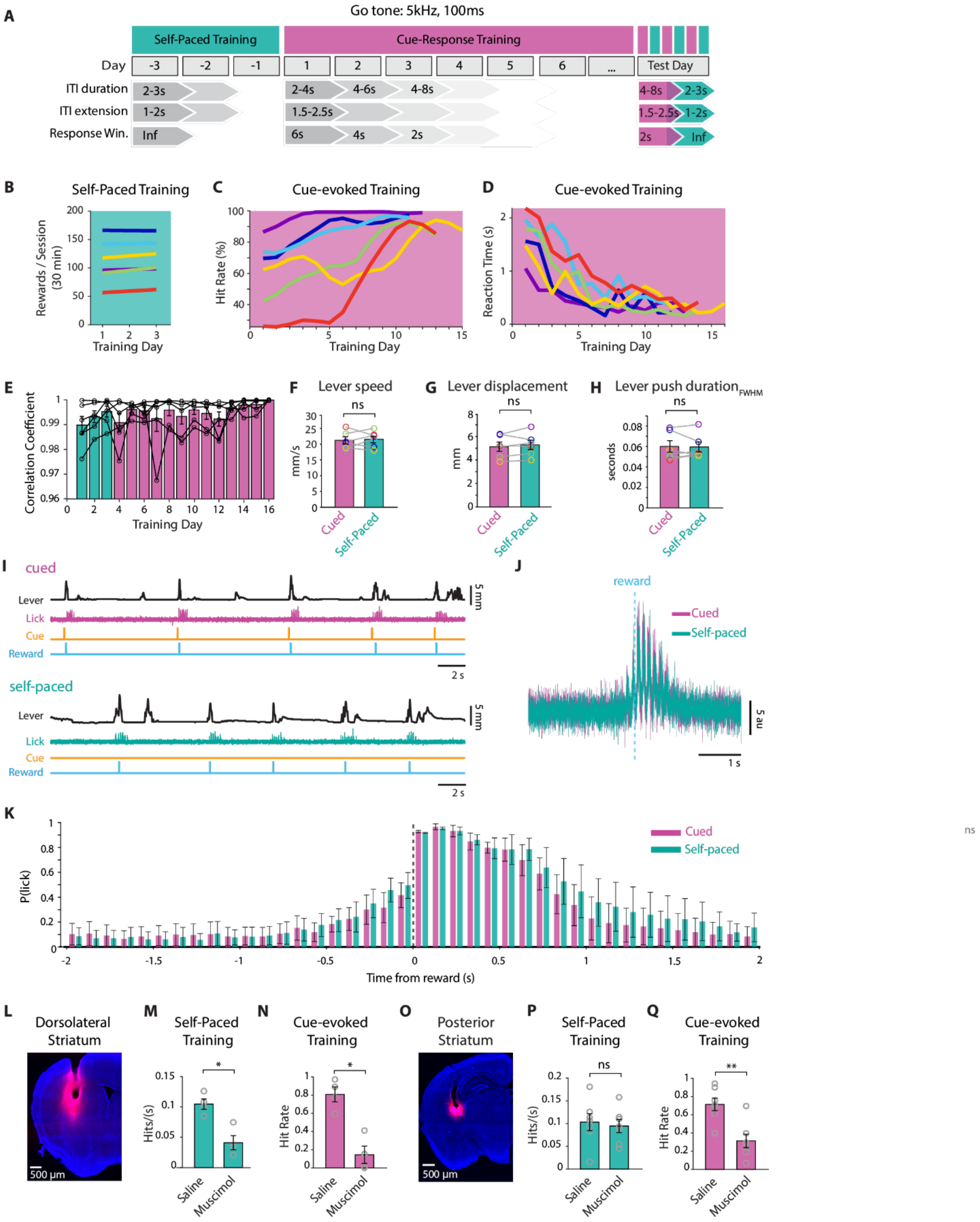

Fig. S1: Learning of the cue-evoked/self-paced lever switching task. (A) Schematic of the training paradigm and timeline for acquisition of the switching task. **(B)** The number of rewards per session during self-paced training, plotted for all 6 mice. **(C)** The performance (plotted as hit rate) on each day cue-evoked training of all 6 mice. (D) The reaction time on each day of cue-evoked training of all 6 mice. **(E)**Average lever press trajectory correlation coefficient across all training sessions for each

individual animal, showing that lever presses become more correlated (stereotypical) over time. Bars are mean ± sem. **(F)** The peak lever speed during the rewarded pushes was not significantly different between cued and self-paced blocks (cued = 21.2 ± 1.1 mm/s; self-paced = 21.5 ± 1.1 mm/s, n = 6; Wilcoxon signed rank test: ns $p > 0.05$). **(G)** The maximum lever displacement was not significantly different between cued and self-paced blocks (cued = 5.12 ± 0.39 mm; self-paced = 5.29 ± 0.40 mm, n = 6; Wilcoxon signed rank test: ns $p > 0.05$). **(H)** The time is took to reach the FWHM (full-width half max) of the displacement was not significantly different between cued and self-paced blocks (cued = 0.06 ± .005 s; self-paced = 0.06 ± .006 s, n = 6; Wilcoxon signed rank test: ns $p > 0.05$). **(I)** Example traces from a mouse showing lick trace that corresponds to rewarded lever presses in cued (top) and self-paced (bottom) blocks. **(J)** For mouse in I, average lick trace in cued (magenta) and self-paced (teal) lever presses. **(K)** Histogram showing the probability of licking across time bins in cued (magenta) and self-paced (teal) blocks. These distributions were not significantly different (two-way ANOVA: $p = 0.11$). **(L)** Histological image showing muscimol injection in the dorsolateral striatum. **(M)** Hit rate for self-paced training was significantly reduced by muscimol injection in the "anterior striatum," or DLS (saline: 0.10 ± 0.009 hits/s; muscimol 0.04 ± 0.01 hits/s; n = 4, Mann-Whitney U test: *$p < 0.05$). Bar plots are mean ± sem. **(N)** Hit rate during cue-evoked training was significantly affected by muscimol injection in the DLS (saline: 0.81 ± 0.08; muscimol 0.19 ± 0.10 hits/s; n = 4, Mann-Whitney U test: *$p < 0.05$). Bar plots are mean ± sem. **(O)** Histological image showing muscimol injection in the "tail," or posterior striatum. **(P)** Hit rate for self-paced training was not affected by muscimol injection in the posterior striatum (saline: 0.10 ± 0.02 hits/s; muscimol 0.09 ± 0.01 hits/s, n = 7, Mann-Whitney U test: ns $p > 0.05$). Bar plots are mean ± sem. **(Q)** Hit rate during cue-evoked training was significantly affected by muscimol injection in the posterior striatum (saline: 0.71 ± 0.07; muscimol 0.31 ± 0.07 hits/s; n = 7, Mann-Whitney U test: **$p < 0.01$). Bar plots are mean ± sem.

.

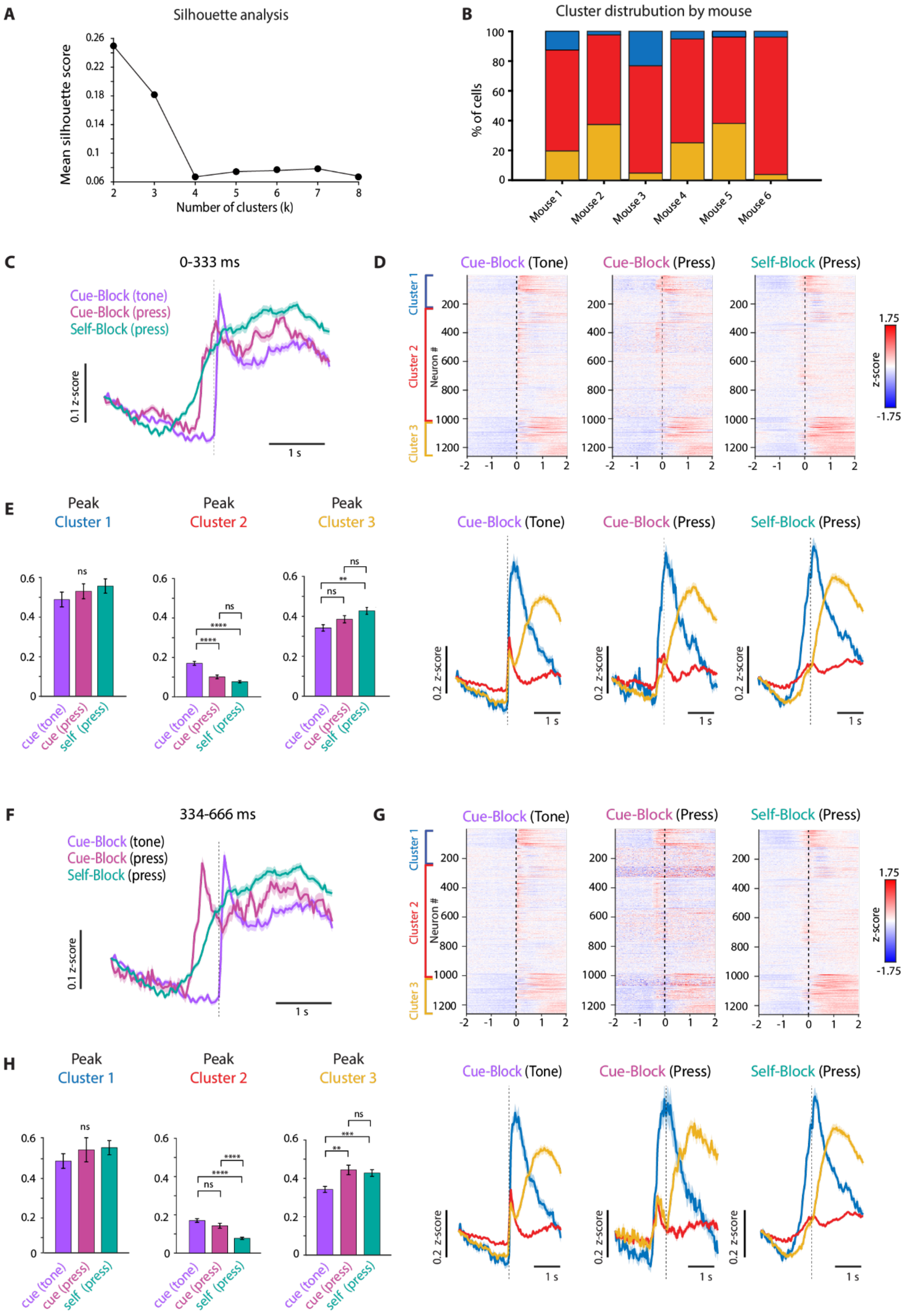

A
Silhouette analysis
Mean silhouette score
Number of clusters (k)
B
Cluster distrubution by mouse
% of cells
Mouse 1
Mouse 2
Mouse 3
Mouse 4
Mouse 5
Mouse 6
C
0-333 ms
Cue-Block (tone)
Cue-Block (press)
Self-Block (press)
0.1 z-score
1 s
D
Cue-Block (Tone)
Cue-Block (Press)
Self-Block (Press)
Cluster 1
Cluster 2
Cluter 3
Neuron #
z-score
E
Peak Cluster 1
Peak Cluster 2
Peak Cluster 3
cue (tone)
cue (press)
self (press)
ns
****
**
0.2 z-score
F
334-666 ms
G
H
***

Fig. S2: Separation of the trials by response time. (A) Mean silhouette score vs. number of clusters (k), indicating that k = 2–3 provides the best clustering structure. **(B)** Proportion of neurons assigned to each cluster for each animal, illustrating variability in cluster composition across mice. **(C)** Grand average z-scored delta F/F responses of all SPNs (n = 1261) for trials with reaction times between 0–333 ms) for cued blocks aligned to the tone (purple), cued blocks aligned to the press (magenta) and self-paced blocks (teal). **(D)** Top: Raster plots of z-scored delta F/F responses of all neurons for trials with 0–333 ms reaction times, organized by cluster with cluster 1 (blue) on top, cluster 2 (red) in the middle and cluster 3 (yellow) at the bottom. Left is aligned to cue onset in cued blocks, middle is aligned to lever press in cued blocks and right is aligned to lever press in self-paced blocks. Bottom: Corresponding grand average z-scored deltaF/F response for each cluster, aligned to the cue or press, as in the rasters above. **(E)** Left: quantification of the peak deltaF/F z-scored response of cluster 1, aligned to the cue (purple), the press in cued blocks (magenta) and the press in self-evoked blocks (teal). The difference between these groups was not significant (z-scored peak responses: cue aligned = 0.49 ± 0.04, cued, press aligned = 0.53 ± 0.04, self, press aligned = 0.56 ± 0.04, n = 118, One-way ANOVA: p = 0.46 ns p > 0.05). Middle: quantification of the peak deltaF/F z-scored response of cluster 2, aligned to the cue (purple), press in cued blocks (magenta) and press in self-evoked blocks (teal). This group showed the largest peak response to the cue and press in cued trials, which were both significantly larger than the peak response in the self-paced trials (z-scored peak responses: cue aligned = 0.17 ± 0.01, cued, press aligned = 0.10 ± 0.01, self, press aligned = 0.06 ± 0.01, n = 868, One-way ANOVA: $p < 1 \times 10^{-10}$ with Tukey-Kramer post hoc test: ****p < .0001, ns p > 0.05. Right: quantification of the peak deltaF/F z-scored response of cluster 3, aligned to the cue (purple), press in cued blocks (magenta) and press in self-evoked blocks (teal). This group showed the the largest peak response when aligned to the press in both cued and self-paced blocks (z-scored peak responses: cue aligned = 0.34 ± 0.02, cued, press aligned = 0.39 ± 0.02, self, press aligned = 0.43 ± 0.02, n = 275, One-way ANOVA: p = 0.002 with Tukey-Kramer post hoc test: **p < 0.01, ns p > 0.05. **(F)** As in (A) but for trials in which reaction times were between 334 – 666 ms. **(G)** As in B, but for trials in which reaction times were between 334 – 666 ms. **(H)** Left: quantification of the peak deltaF/F z-scored response of cluster 1, aligned to the cue (purple), press in cued blocks (magenta) and press in self-evoked blocks (teal). The difference between these groups was not significant (z-scored peak responses: cue aligned = 0.49 ± 0.04, cued, press aligned = 0.55 ± 0.06, self, press aligned = 0.56 ± 0.04, n = 118, One-way ANOVA: p = 0.53, ns p > 0.05). Middle: quantification of the peak deltaF/F z-scored response of cluster 2, aligned to the cue (light teal), press in cued blocks (magenta) and press in self-evoked blocks (teal). This group again showed the largest peak response to the cue and press in cued trials, which were significantly larger than the peak response in the self-paced trials (z-scored peak responses: cue aligned = 0.17 ± 0.01, cued, press aligned = 0.14 ± 0.01, self, press aligned = 0.06 ± 0.01, n = 868, One-way ANOVA: $p = 1.3 \times 10^{-10}$ with Tukey-Kramer post hoc test: ****p < .0001, ns p > 0.05. Right: quantification of the peak deltaF/F z-scored response of cluster 3, aligned to the cue (purple), press in cued blocks (magenta) and press in self-evoked blocks (teal). This group showed the largest peak response to the press in cued and self-paced trials, which were significantly larger than the peak response in the cue aligned trials (z-scored peak responses: cue aligned = 0.34 ± 0.02, cued, press aligned = 0.44 ± 0.02, self, press aligned = 0.43 ± 0.02, n = 275, One-way ANOVA: p = 0.005 with Tukey-Kramer post hoc test: **p < 0.01, ***p < 0.001, ns p > 0.05. Shaded area in all line plots is ± SEM. Bar plots are mean ± sem.

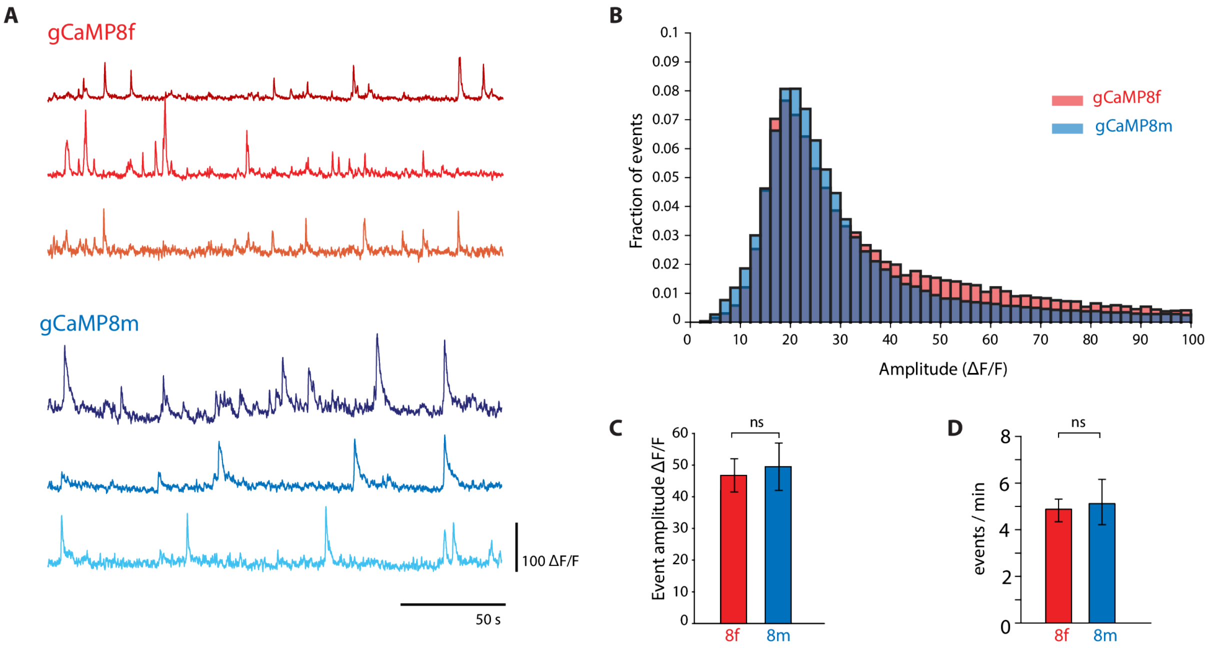

Fig. S3: Event amplitude and rate in GCaMP8f vs. GCaMP8m animals. (A) Example ΔF/F traces from neurons expressing GCaMP8f (top, red) and GCaMP8m (bottom, blue), illustrating comparable event dynamics across indicators. **(B)** Distribution of event amplitudes (ΔF/F) for GCaMP8f (red) and GCaMP8m (blue) neurons. While amplitude distributions exhibited clear structure across bins ($p < 0.001$), we observed no significant effect of sensor type ($p = 1.00$) nor sensor-by-bin interaction ($p = 0.97$), indicating similar amplitude distributions between 8f and 8m. **(C)** Mean event amplitude for GCaMP8f vs. GCaMP8m animals was not significantly different (deltaF/F z-score: 8f = 47.5, n = 322; 8m = 49.8, n = 939 Student's t-test: $p = 0.12$, ns $p > 0.05$). **(D)** Event rate (events/min) for GCaMP8f and GCaMP8m animals was not significantly different (8f = 5.1 events/min, n = 322; 8m = 4.9 events/min, n = 939; Student's t-test: $p = 0.25$, ns $p > 0.05$). Bar plots are data ± SEM.

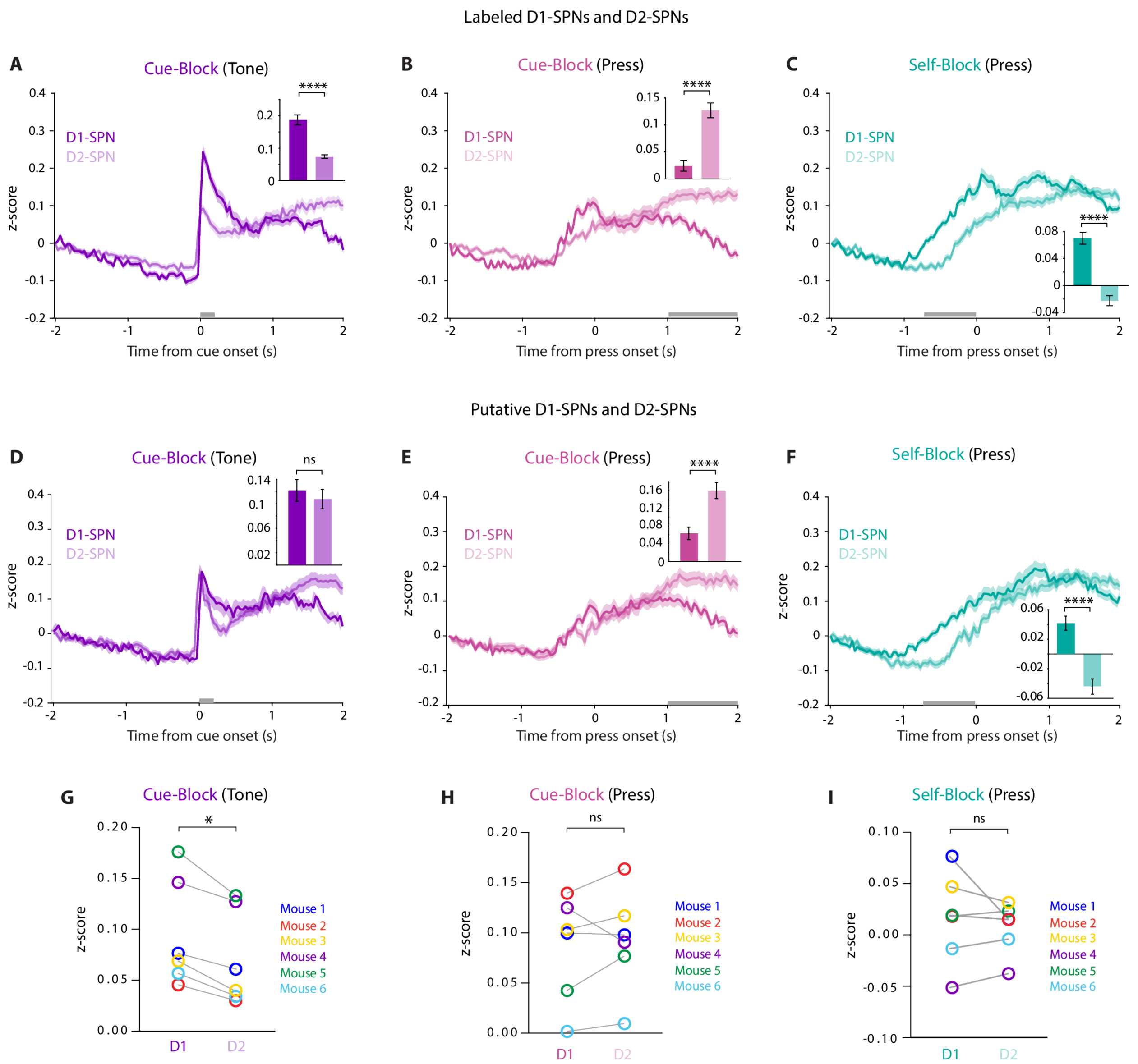

Fig. S4. D1- and D2-SPN response differences in labeled and putative neurons with animal-level comparisons. (A) Average population activity of labeled (i.e. tdTomato positive) D1-SPNs (n = 225; dark purple) and labeled D2-SPNs (n = 281; light purple) aligned to cue presentation. Inset: Mean z-scored deltaF/F activity 0-200ms (indicated by grey bar) after the cue onset was significantly higher in D1-SPNs (mean z-score 0-200 ms from cue onset: D1 = 0.19 ± 0.02, D2 = 0.07 ± 0.01, Student's t-test: $p = 2.1 \times 10^{-9}$, ****$p < 0.0001$). **(B)** Average population activity of labeled (i.e. tdTomato positive) D1-SPNs (n = 225; dark magenta) and labeled D2-SPNs (n = 281; light magenta) aligned to lever press in cued blocks. Inset: Mean z-scored deltaF/F activity 1-2 s after the press onset (indicated by grey bar) was significantly higher in D2-SPNs (mean z-score 1-2 s from cue onset: D1 = 0.02 ± 0.01, D2 = 0.13 ± 0.01, Student's t-test: $p = 2.3 \times 10^{-9}$, ****$p < 0.0001$). **(C)** Average population activity of labeled (i.e. tdTomato positive) D1-SPNs (n = 225; dark teal) and labeled D2-SPNs (n = 281; light teal) aligned to lever press in self-evoked blocks. Inset: Mean z-scored deltaF/F activity -.75 to 0 s relative to press (indicated by grey bar) onset was significantly higher in D1-SPNs (mean z-score -750-0 ms from press onset in self-paced block: D1 = 0.07 ± 0.01, D2 = -0.02 ± 0.01, p = 0.006, Student's t-test: $p = 4.3 \times 10^{-15}$, ****$p < 0.0001$). **(D)** Average population activity of putative (i.e. tdTomato negative) D1-SPNs (n = 313; dark purple) and putative D2-SPNs (n = 248; light purple) aligned to cue presentation. Inset: Mean z-scored deltaF/F activity 0-200ms (indicated by grey bar) after the cue onset was not significantly different (mean z-score 0-200 ms from cue onset: D1 = 0.12 ± 0.02, D2 = 0.11 ± 0.02, Student's t-test: p = 0.55, ns $p > 0.05$). **(E)** Average population activity of putative (i.e. tdTomato negative) D1-SPNs (n = 313; dark magenta) and putative D2-SPNs (n = 248; light magenta) aligned to press in cued blocks. Inset: Mean z-scored deltaF/F activity 1- 2 s (indicated by grey bar) after the cue onset was significantly higher in D2-SPNs (mean z-score 1-2 s from cue onset: D1 = 0.06 ± 0.01, D2 = 0.16 ± 0.02, Student's t-test: $p = 3.7 \times 10^{-5}$, ****$p < 0.0001$). **(F)** Average population activity of putative (i.e. tdTomato negative) D1-SPNs (n = 313; dark teal) and putative D2-SPNs (n = 248; light teal) aligned to press in self-paced blocks. Inset: Mean z-scored deltaF/F activity -.75 to 0 s relative to press (indicated by grey bar) after the cue onset was significantly higher in D1-SPNs (mean z-score -750-0 ms from press onset in self-paced block: D1 = 0.04 ± 0.01, D2 = -0.04 ± 0.01, p = 0.006, Student's t-test: $p = 4.3 \times 10^{-9}$, ****$p < 0.0001$). **(G)** D1–D2 comparisons at the level of individual animals (including both labeled and putative

neurons) for the corresponding time window in the cue-aligned plots above in (D). Each point represents the mean response for a single mouse, with lines connecting D1 and D2 values within the same animal (two-sided Wilcoxon signed-rank test: *$p < 0.05$). **(H)** D1–D2 comparisons at the level of individual animals (including both labeled and putative neurons) for the corresponding time window in the press-aligned plots in cued blocks above in (E). Each point represents the mean response for a single mouse, with lines connecting paired D1 and D2 values within the same animal (two-sided Wilcoxon signed-rank test: ns, $p > 0.05$). **(I)** D1–D2 comparisons at the level of individual animals (including both labeled and putative neurons) for the corresponding press-aligned time window in self-paced blocks above in (F). Each point represents the mean response for a single mouse, with lines connecting paired D1 and D2 values within the same animal (two-sided Wilcoxon signed-rank test: ns, $p > 0.05$). Shaded bars in all line plots are ± SEM.

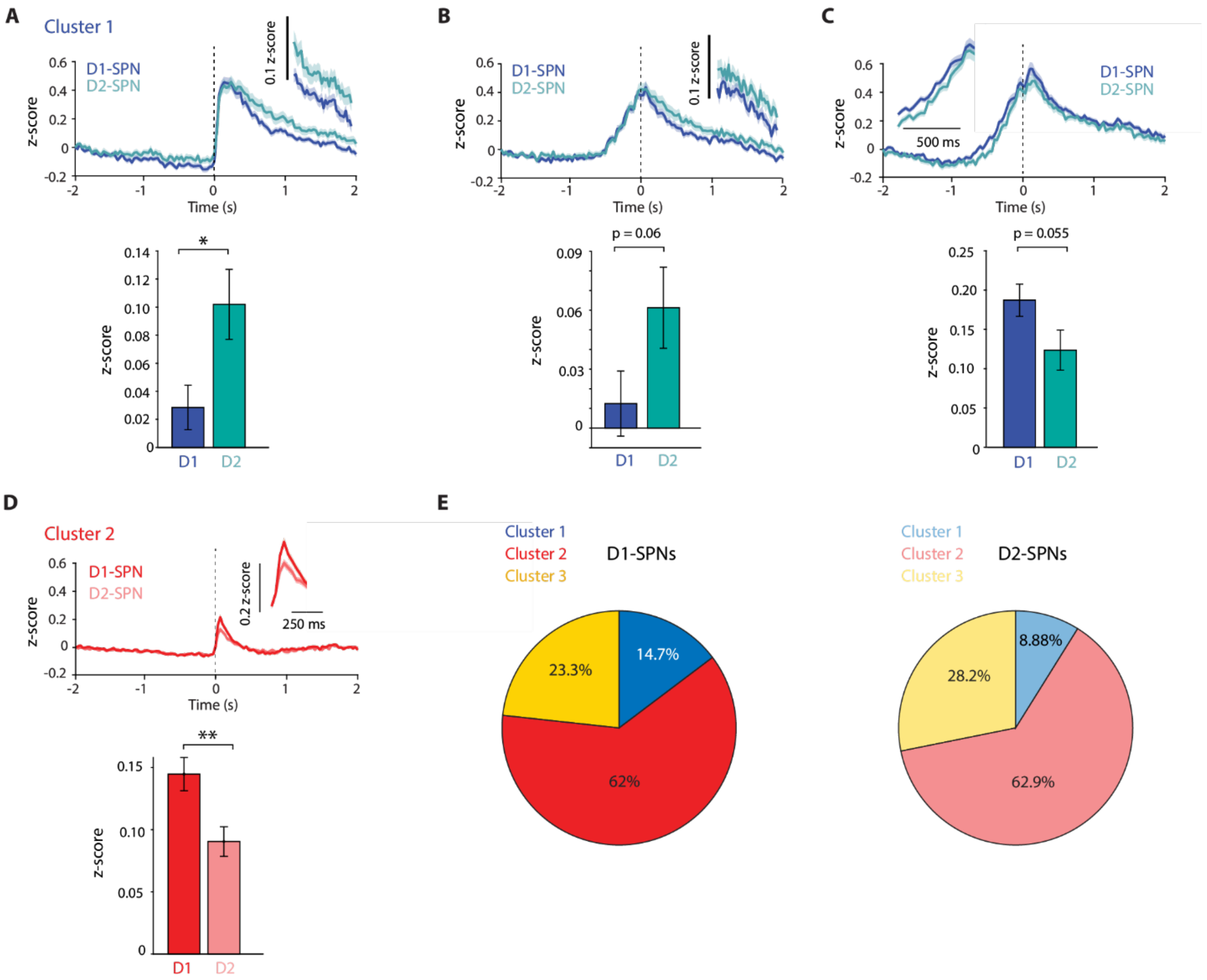

Fig. S5. Quantification of average D1 and D2 responses by cluster. (A) Z-scored ΔF/F responses of cluster 1 D1-SPNs (n = 80; dark blue) and D2-SPNs (n = 47; teal) aligned to the tone in the cued blocks with inset showing area quantified in bar graph below (1-2 s post press). D2-SPNs in this cluster showed a significantly larger response than D1-SPNs in this time window (mean z-score 1 -2 s from cue onset aligned to press in self-paced block: D1 = 0.03 ± 0.02, D2 = 0.10 ± 0.02, Student's t-test: p = 0.01, *p < 0.05). **(B)** Z-scored ΔF/F responses of cluster 1 D1-SPNs (n = 80; dark blue) and D2-SPNs (n = 47; teal) aligned to the press in the cued blocks with inset showing area quantified in bar graph below, between 1 and 2 s post press. D2-SPNs in this cluster showed a higher response than D1-SPNs in this time window, although this difference was not significant (mean z-score 1 -2 s from cue onset aligned to press in self paced block: D1 = 0.01 ± 0.02, D2 = 0.06 ± 0.02, Student's t-test: p = 0.06). **(C)** Z-scored ΔF/F responses of cluster 1 D1-SPNs (n = 80; dark blue) and D2-SPNs (n = 47; teal) aligned to the press in the cued blocks with inset showing area quantified in bar graph below, between -750 – 0 ms before the press. D1-SPNs in this cluster showed a higher response than D2-SPNs in this time window, although this difference was not significant (mean z-score -750 – 0 ms from cue onset aligned to press in self paced block: D1 = 0.19 ± 0.02, D2 = 0.12 ± 0.03, Student's t-test: p = 0.055). **(D)** Z-scored ΔF/F responses of cluster 2 D1-SPNs (n = 333; dark red) and D2-SPNs (n = 333; light red) aligned to the tone in the cued blocks with inset showing area quantified in bar graph below, between 0 –200 ms after the tone onset. D1-SPNs in this cluster showed a significantly higher response than D2-SPNs in this time window (mean z-score 0 – 200 ms from cue onset aligned to press in self paced block: D1 = 0.14 ± 0.01, D2 = 0.09 ± 0.01, Student's t-test: **p < 0.01). **(E)** Pie charts showing proportion of cluster 1, 2 and 3 neurons in D1-SPN (left, darker colors) and D2-SPN (right, lighter colors) subpopulations. Shaded area in all line plots is ± SEM. Bar plots are data ± SEM.

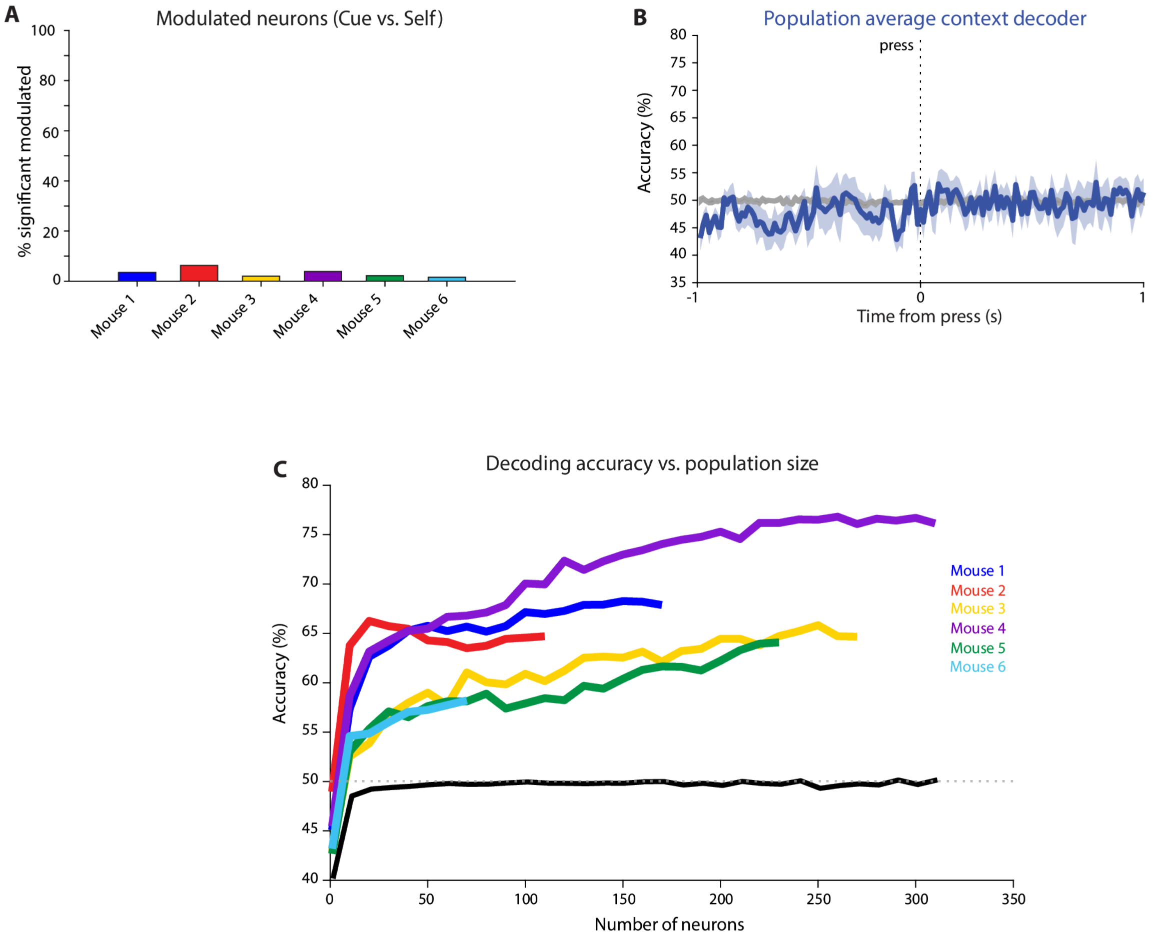

Fig. S6. Population-level context decoding across animals. (A) Percentage of neurons significantly modulated by context (cue vs. self) for each animal. **(B)** A support vector machine classifier trained on the average population activity of each trial ("Population average context decoder") could not predict context. Gray plot is the prediction on shuffled data. **(C)** Decoding accuracy as a function of population size for each animal. Neurons were sequentially added to the decoder, demonstrating that decoding performance improves with increasing population size. Each line represents one animal, and the dashed line indicates chance-level performance. Black indicates the shuffled distribution generated by pooling neurons across animals and randomly permuting trial identities.

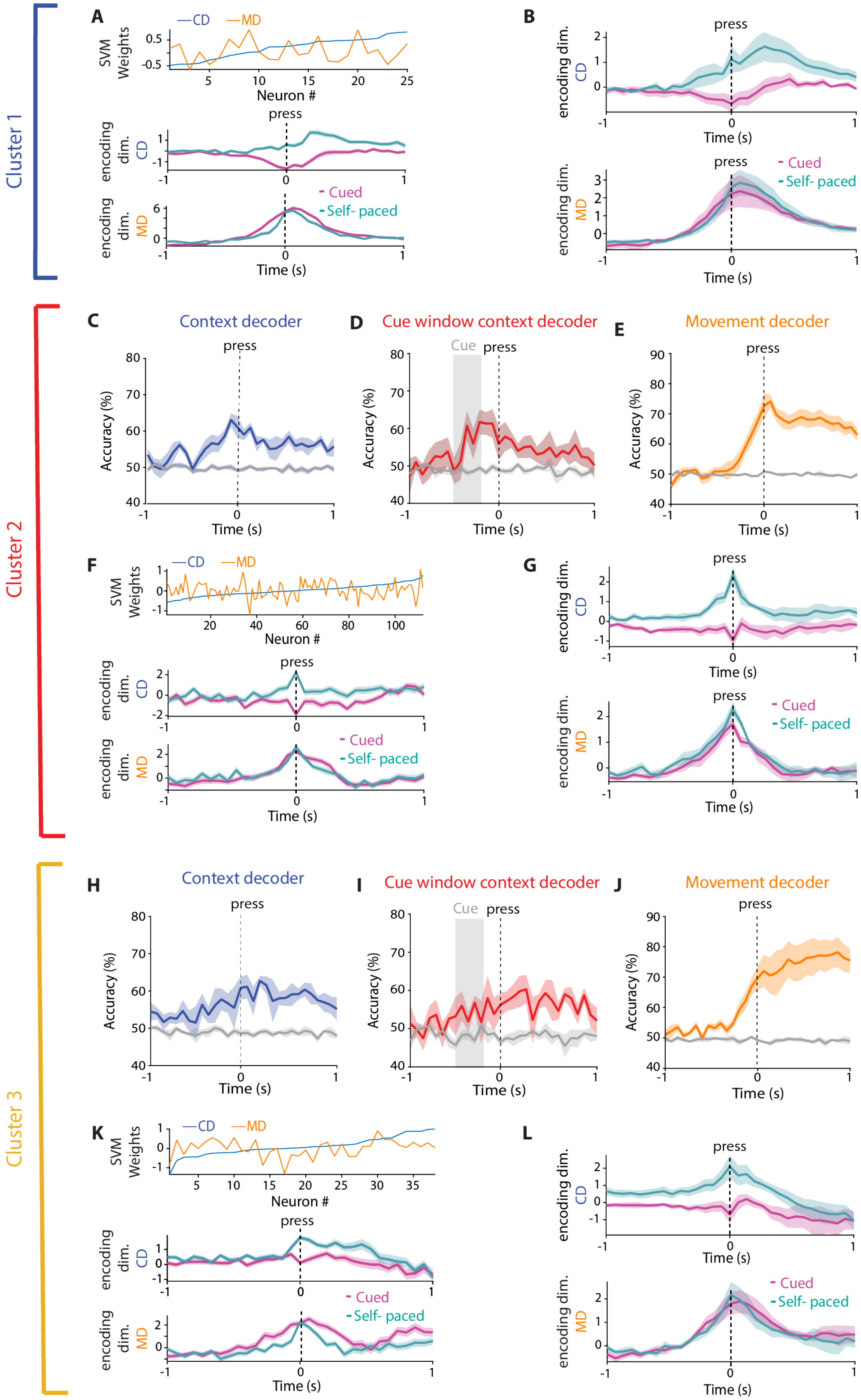

Cluster 1
A
SVM Weights
CD
MD
Neuron #
encoding dim. CD
press
encoding dim. MD
Cued
Self- paced
Time (s)
B
Cluster 2
C
Context decoder
Accuracy (%)
D
Cue window context decoder
Cue
E
Movement decoder
F
G
Cluster 3
H
I
J
K
L

Fig. S7. Population decoding by cluster. (A) For Cluster 1 neurons, Top: Neuron weights from the “Context decoder” (cue vs. self-paced) plotted in order of magnitude (blue), with overlaid weights from the movement decoder (press vs. baseline, orange) showing distinct, uncorrelated contributions to each classification axis. Population activity from an example mouse projected onto SVM-defined axes shows trial-type-specific separation peaking at movement onset (middle) and shared motor-related dynamics across conditions (bottom). **(B)** Same as A middle and bottom, for all Cluster 1 neurons. **(C)** For cluster 2 neurons a the “Context decoder” trained on 90% of the data and tested on the remaining 10% successfully predicted whether lever presses were cue-evoked or self-initiated based on activity, indicating that this subpopulation carries distinguishable population activity patterns across trial types. Gray plot is the prediction on shuffled data. **(D)** For cluster 2 neurons, classification accuracy by the “Cue window context decoder” rose above chance during the cue period (−495 to −165 ms before the press in matched-latency trials), indicating that this subpopulation reliably encoded movement type prior to lever press onset. Gray plot is the prediction on shuffled data. **(E)** For cluster 2 neurons, the classification accuracy by the “Cue window context decoder” rose above chance during the cue period performed above chance before movement onset, indicating that this ensemble contributes to a shared preparatory neural dimension across both action types. Gray plot is the prediction on shuffled data. **(F)** Same as (A), for cluster 2 neurons. **(G)** Same as (B), for cluster 2 neurons. **(H)** Same as (C), for cluster 3 neurons. **(I)** Same as (D), for cluster 3 neurons. **(J)** Same as (E), for cluster 3 neurons. **(K)** Same as (F), for cluster 3 neurons. **(L)** Same as (G), for cluster 3 neurons. Shaded area in all line plots is ± SEM.

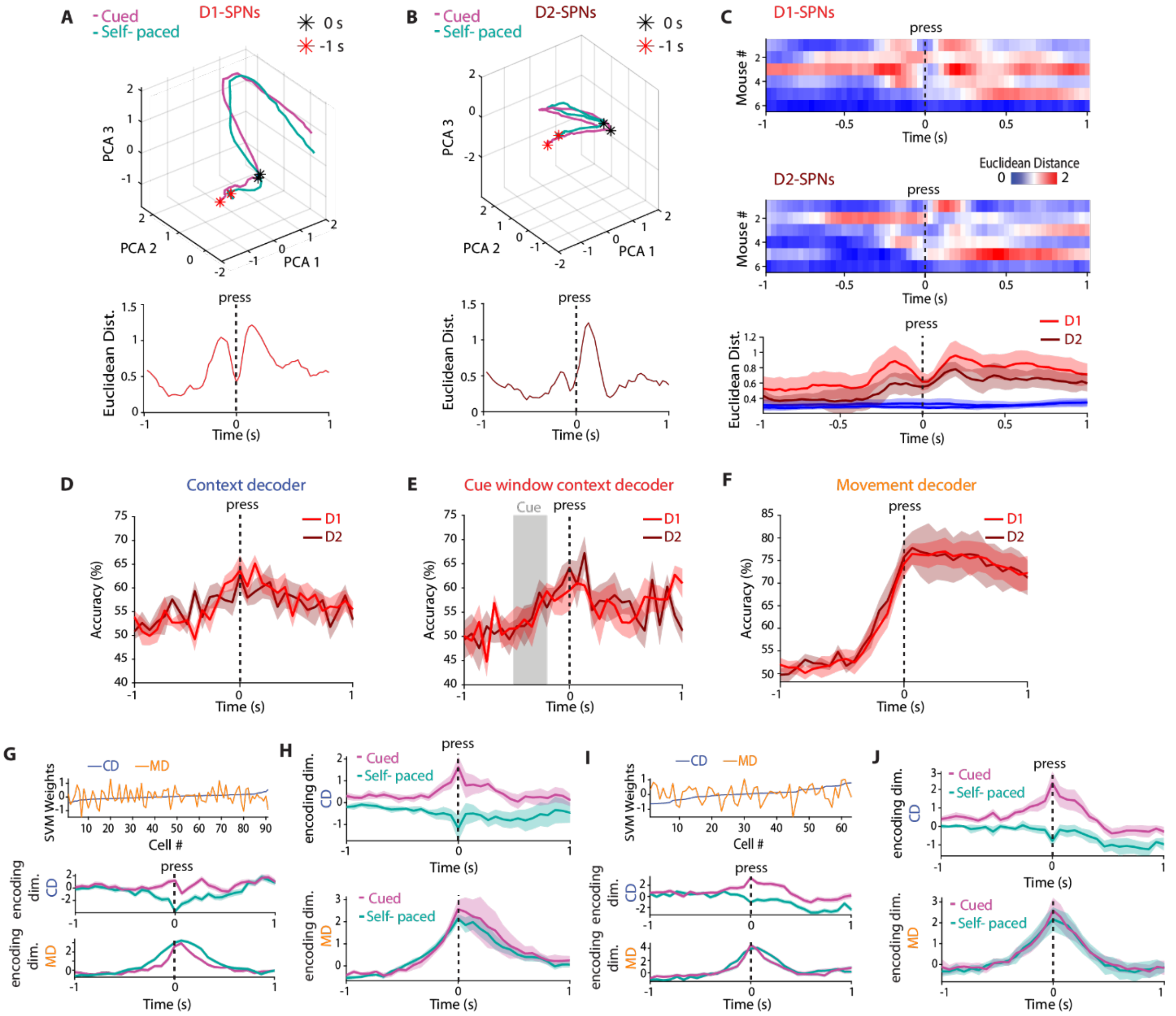

Fig. S8. Context decoding by cell type. (A) Top: Principal component analysis of D1-SPN population activity shows that neural trajectories for cue-evoked (teal) and self-paced (magenta) trials in an example mouse. Black asterisk is time of the press/reward delivery and red asterisk is 1 second before that. Bottom: distance between neural trajectories shown above versus time. **(B)** Same as (A), for D2-SPNs. **(C)** Euclidean distance between neural trajectories for cue-evoked and self-paced trials is shown for individual mice in D1-SPN (top) and D2-SPN (middle) populations. Bottom: The average of all D1-SPNs in all mice (red) and D2-SPNs (dark red) in all mice compared to shuffled data (blue). **(D)** Support vector machine ("Context decoder") classifier trained on 90% of the data and tested on the remaining 10% successfully predicted whether lever presses were cue-evoked or self-initiated based on activity in both D1-SPNs (red) and D2-SPNs (dark red) populations, indicating that both cell types carry distinguishable population activity patterns across trial types. **(E)** SVM ("Cue window context decoder") classification accuracy rose above chance during the cue period (−495 to −165 ms before the press) for matched-latency trials for both D1-SPNs (red) and D2-SPNs (dark red). **(F)** For D1-SPNs (red) and D2-SPNs (dark red) neurons, the classifier distinguishing lever presses from baseline ("Movement decoder") performed above chance before movement onset, indicating that this ensemble contributes to a shared preparatory neural dimension across both action types. **(G)** For D1-SPNs, top: weights from the "Context decoder (CD)" (press vs. baseline) are plotted in order of magnitude (blue), with overlaid weights from the "Movement decoder (MD)" (cue vs. self-paced, orange) showing distinct and uncorrelated contributions to each classification axis. Projections of D1-SPN activity onto these SVM-defined axes reveal trial-type-specific separation peaking at movement onset (middle) and shared motor-related dynamics across conditions (bottom). **(H)** Same as G middle and bottom, for all D1-SPNs. **(I)** Same as G, for D2-SPNs. **(J)** Same as (I) middle and bottom, for all D2-SPNs. Shaded bars in all line plots are ± SEM.
